# A Comprehensive Drift-Adaptive Framework for Sustaining Model Performance in COVID-19 Detection From Dynamic Cough Audio Data: Model Development and Validation

**DOI:** 10.2196/66919

**Published:** 2025-06-03

**Authors:** Theofanis Ganitidis, Maria Athanasiou, Konstantinos Mitsis, Konstantia Zarkogianni, Konstantina S Nikita

**Affiliations:** 1 School of Electrical and Computer Engineering National Technical University of Athens Athens Greece; 2 Faculty of Science and Engineering Maastricht University Maastricht The Netherlands; 3 Viterbi School of Engineering University of Southern California Los Angeles, CA United States

**Keywords:** COVID-19 detection, machine learning, model degradation, data distribution shift, maximum mean discrepancy, unsupervised domain adaptation, active learning

## Abstract

**Background:**

The COVID-19 pandemic has highlighted the need for robust and adaptable diagnostic tools capable of detecting the disease from diverse and continuously evolving data sources. Machine learning models, particularly convolutional neural networks, are promising in this regard. However, the dynamic nature of real-world data can lead to model drift, where the model’s performance degrades over time, as the underlying data distribution changes due to evolving disease characteristics, demographic shifts, and variations in recording conditions. Addressing this challenge is crucial to maintaining the accuracy and reliability of these models in ongoing diagnostic applications.

**Objective:**

This study aims to develop a comprehensive framework that not only monitors model drift over time but also uses adaptation mechanisms to mitigate performance fluctuations in COVID-19 detection models trained on dynamic cough audio data.

**Methods:**

Two crowdsourced COVID-19 audio datasets, namely COVID-19 Sounds and Coswara, were used for development and evaluation purposes. Each dataset was divided into 2 distinct periods, namely the development period and postdevelopment period. A baseline convolutional neural network model was initially trained and evaluated using data (ie, coughs from COVID-19 Sounds and shallow coughs from Coswara dataset) from the development period. To detect changes in data distributions and the model’s performance between these periods, the maximum mean discrepancy distance was used. Upon detecting significant drift, a retraining procedure was triggered to update the baseline model. The study explored 2 model adaptation approaches, unsupervised domain adaptation and active learning, both of which were comparatively assessed.

**Results:**

The baseline model achieved an area under the receiver operating characteristic curve of 69.13% and a balanced accuracy of 63.38% on the development test set of the COVID-19 Sounds dataset, while for the Coswara dataset, the corresponding values were 66.8% and 61.64%. A decline in performance was observed when the model was evaluated on data from the postdevelopment period, indicating the presence of model drift. The application of the unsupervised domain adaptation approach led to performance improvement in terms of balanced accuracy by up to 22% and 24% for the COVID-19 Sounds and Coswara datasets, respectively. The active learning approach yielded even greater improvement, corresponding to a balanced accuracy increase of up to 30% and 60% for the 2 datasets, respectively.

**Conclusions:**

The proposed framework successfully addresses the challenge of model drift in COVID-19 detection by enabling continuous adaptation to evolving data distributions. This approach ensures sustained model performance over time, contributing to the development of robust and adaptable diagnostic tools for COVID-19 and potentially other infectious diseases.

## Introduction

### Background

The rapid spread of SARS-CoV-2 and its associated disease, COVID-19, has created a pressing need for accurate and timely diagnostic tools. Traditional diagnostic methods, such as polymerase chain reaction tests, while reliable, often involve invasive procedures and can be time consuming. Consequently, there is a growing interest in developing additional diagnostic approaches that are noninvasive, affordable, scalable, and capable of delivering swift results [1].

Deep learning models have demonstrated exceptional capabilities across various domains, including medical diagnostics [2-8] and epidemiological surveillance [9]. Studies have illuminated the potential of harnessing deep learning techniques for analyzing diverse data sources, such as clinical and biological biomarkers, computed tomography scan imagery, and clinical characteristics, to predict the severity and progression of COVID-19 [10-13]. In recent studies, the analysis of cough sounds has shown potential as a noninvasive modality for COVID-19 detection [14-16]. Leveraging the power of deep learning, these models can extract crucial information from acoustic characteristics, aiding in the early identification of individuals who are infected.

However, in practice, the performance of deep learning models tends to decline during deployment and shows further deterioration over time. This phenomenon, commonly known as model degradation, can be attributed to various factors [17]. One contributing factor is the limited representation of the training data, which fails to capture the complexity of the problem space adequately. Consequently, the model exhibits unexpected behavior when confronted with input samples lying outside the distribution of training examples [18,19]. Another significant factor is the dynamic nature of the system’s environment, which undergoes continuous changes over time [18], posing challenges for a single model to maintain accurate predictions consistently. This factor is particularly critical in the context of COVID-19, given the rapid and unpredictable changes due to several reasons, including the emergence of new viral strains.

The literature refers to these 2 aforementioned factors as concept drift, which is the phenomenon where the input data and their relationship to the labels undergo changes over time. Numerous attempts have been made in the past decade to precisely define concept drift [17,20-23]. In this paper, the definition from the study by Lu et al [22] is adopted, which states that concept drift occurs when either the data distribution changes, the underlying relationship between the input and output changes, or both change.

In the context of respiratory diseases, several previous studies have acknowledged the limitations posed by concept drift in crowdsourced respiratory datasets, highlighting the variability introduced by self-reported ground truth labels, the lack of clinical validation, and the evolving symptomatology of different variants of SARS-CoV-2 [24]. These factors contribute to performance degradation and uncertainty in the extracted features. In addition, dataset biases due to demographic imbalances and variations in symptom severity further complicate model reliability over time [25].

Researchers have recognized the importance of addressing these challenges and have focused on learning in nonstationary environments [26] and mitigating the impact of concept drift [27-30]. Research studies have stressed the importance of integrating a model degradation detector within the learning framework [31] that assesses and tracks the system’s performance after deployment to effectively manage the degradation in prediction accuracy. The level of degradation in the model performance serves as an indicator for detecting concept drift within the system. By incorporating these detection components, deep learning systems develop resilience against environmental changes, thereby mitigating the performance degradation of predictive models in this ever-changing setting.

Because the presence of concept drift between training data and real postdevelopment data impedes the performance of deep learning models on out-of-distribution samples [27], applying the model on new data may necessitate adaptation. Automatic methods have emerged to tackle these challenges; however, collecting large-scale labeled datasets for different populations, emerging virus variants, or new pandemics is an arduous task. When working with limited data, it is often necessary to use more cost-efficient deep learning methodologies, such as unsupervised domain adaptation (UDA) and active learning (AL).

Domain adaptation is a technique used to address the limited generalization ability of predictive models when the training and testing data come from different distributions [32]. The goal is to adapt a model trained on a source domain to perform well on a target domain. This involves minimizing the distribution gap between the domains through learning domain-invariant features [33,34], weighing samples based on similarities [35], or using model-based techniques such as domain adversarial networks [36,37]. These approaches can improve model generalization in real-world scenarios with varying data distributions, as they enable learning from labeled data in the development set, which refers to the past, and applying this knowledge to solve tasks on postdevelopment unlabeled data.

AL, by contrast, is a machine learning approach where informative samples from a large, unlabeled dataset are selected and labeled iteratively to train a model. The objective is to minimize the amount of labeled data needed while maximizing the model’s performance [38-40]. A query strategy is selected to determine which unlabeled samples should be labeled. Various strategies exist, such as uncertainty sampling [41,42] or diversity sampling [43]. On the basis of the initially trained model, the chosen query strategy is applied to the unlabeled dataset, identifying the most informative samples based on the selected criterion. These selected samples are labeled either manually by domain experts or through an automated process. The newly labeled samples are incorporated into the labeled dataset, which is used for retraining the model.

Most recent studies focusing on COVID-19 detection based on the use of audio recordings have primarily used supervised deep learning techniques [15,16,44-50]. It is noteworthy that in most of these studies, aspects related to the existence of concept drift and the challenges of model generalization have not been addressed. In the study by Han et al [16], a rather complex model architecture has been presented with a deeper fully connected part, enhancing performance when multiple modalities (cough, voice, and breathing) are used but leading to reduced accuracy when relying solely on cough recordings. While the study by Han et al [16] acknowledges potential biases and dataset limitations, its analysis does not explicitly consider temporal aspects, such as how biases or drifts emerge over time as new data are collected.

Earlier attempts to address data drift issues in the context of respiratory diseases, including COVID-19, have primarily relied on transfer learning techniques to mitigate performance degradation [51,52]. However, these methods often assume that the source and target domains share a strong underlying similarity, which may not hold when data distributions drift significantly, potentially leading to poor performance. In general, the exploration of methods relying on concept drift detection, model degradation detection, UDA, and AL has been limited [29,53]. A few recent approaches have investigated adversarial domain adaptation to enhance model generalizability across datasets. For instance, in the study by Nguyen et al [37], a domain adaptation framework for respiratory symptom detection has been proposed, concentrating on static cross-dataset generalization. AL has also been explored as an efficient strategy for improving model performance while minimizing the labeling burden, particularly in resource-constrained scenarios such as pandemic response. In the study by Wu et al [40], a deep AL framework has been developed for COVID-19 diagnosis from computed tomography scans, using a hybrid sampling strategy to optimize labeling efforts. Thus, UDA and AL approaches appear to be highly promising and well-suited for addressing the continuously evolving nature of pandemics, as they enable the development of reliable models with the potential to address even the emergence of novel virus variants.

### This Study

In this paper, a comprehensive framework is introduced for the diagnosis of infectious diseases, focusing on COVID-19 detection from cough sounds. The framework leverages deep learning models combined with UDA- and AL-based methodologies to monitor and mitigate model degradation and concept drift. The development and evaluation of the proposed framework is demonstrated on the COVID-19 data due to their continuously evolving epidemiological and virological characteristics, arising from the complex interplay among the virus, humans, vaccines, and environments. The maximum mean discrepancy (MMD) [54] distance is first used as a metric to quantify the dissimilarity between temporal data distributions. By monitoring the MMD distance between batches of postdevelopment data and data from the initial development period, the framework detects changes in both the data and the model’s performance while also providing insights into the impact of the pandemic’s evolution on the trained models’ diagnostic accuracy. If concept drift is detected, a retraining process is initiated, including two adaptation methods: (1) a UDA process, which leverages labeled development data along with unlabeled postdevelopment data to align their distributions and adapt the model to novel data instances and (2) an AL strategy, aimed at selecting informative data to include them with their labels in the retraining process. To the best of our knowledge, this is the first work leveraging UDA and AL approaches toward mitigating the impact of evolving data dynamics on model performance for COVID-19 detection, with the ultimate goal of enhancing reliability in COVID-19 detection and potentially across various diverse epidemiological contexts.

## Methods

### Datasets

The COVID-19 Sounds dataset is a collection of respiratory sound recordings associated with COVID-19 infections, which were acquired through a crowdsourcing platform launched in April 2020 [16]. It includes demographic characteristics (ie, age and gender), along with participant-reported information about medical history and symptoms. It also comprises audio clips of voluntary cough, breathing, and voice captured from healthy individuals and individuals with COVID-19. A total of 36,364 participants contributed 75,201 samples to the project. Quality checks were performed on the audio samples to filter out incomplete or noisy recordings [16]. The data were collected in multiple languages, but for this study, the part of the dataset acquired from English-speaking participants [16] was solely considered to avoid language bias, corresponding to 1461 samples, as shown in Table 1.

The Coswara dataset is another crowdsourced database recorded between April 2020 and February 2022, which consists of 9 types of recordings, such as shallow and deep breaths, shallow and heavy coughs, sustained vowel phonation (ie, <ey> as in made, <i> as in beet, and <u:> as in cool), and number counting from 1 to 20 (normal and fast paced) [55]. Alongside this, information on the participants’ COVID-19 infection status, symptoms, comorbidities (if any), gender, age, and broad geographical location is included. In this study, shallow cough recordings were used as the models’ input space. After the exclusion of any missing, corrupted, or silent samples, a total of 72.69% (1996/2746) of samples from the initial set remained for analysis, as shown in Table 1.

**Table 1:** Partition of the used datasets into development and postdevelopment sets.

	COVID-19 Sounds dataset samples, n	Coswara dataset samples, n
Development set	1040	1395
COVID-19–positive samples of the development set	452	165
Postdevelopment set	421	601
COVID-19–positive samples of the postdevelopment set	270	482

^a^Not applicable.

Following the preprocessing approach described in the study by Han et al [16], the cough recordings were normalized, and leading and trailing silence was removed. Mel spectrograms were calculated using a 25-millisecond window size, a 10-millisecond window hop, and 64 mel bins, encompassing frequencies ranging from 125 Hz to 7500 Hz. In addition, within the framework of this study, in order to handle the varying size of the mel spectrograms’ time axis, the 0.9 quantile across all spectrograms was calculated. Subsequently, the spectrograms were either cropped or padded with repeated sections of the spectrogram accordingly. Finally, a sliding window approach was used to extract segments from the spectrogram. The width of the window used was 0.96 seconds, while the window stride length was equal to half of the window’s width (0.48 s). This setting resulted in a mel spectrogram segment with a size of 64 mel bins × 96 frames.

To facilitate model training, the COVID-19 Sounds and Coswara datasets were partitioned based on chronological order into a development set and a postdevelopment set by applying a 70:30 ratio. The development set was further divided into training, validation, and testing subsets, using a 60:20:20 split ratio, respectively. This division ensured that the model was trained on a representative portion of the data and validated and tested on separate subsets, promoting robustness and generalization.

### Proposed Methodological Framework

#### Overview

**Figure 1:**
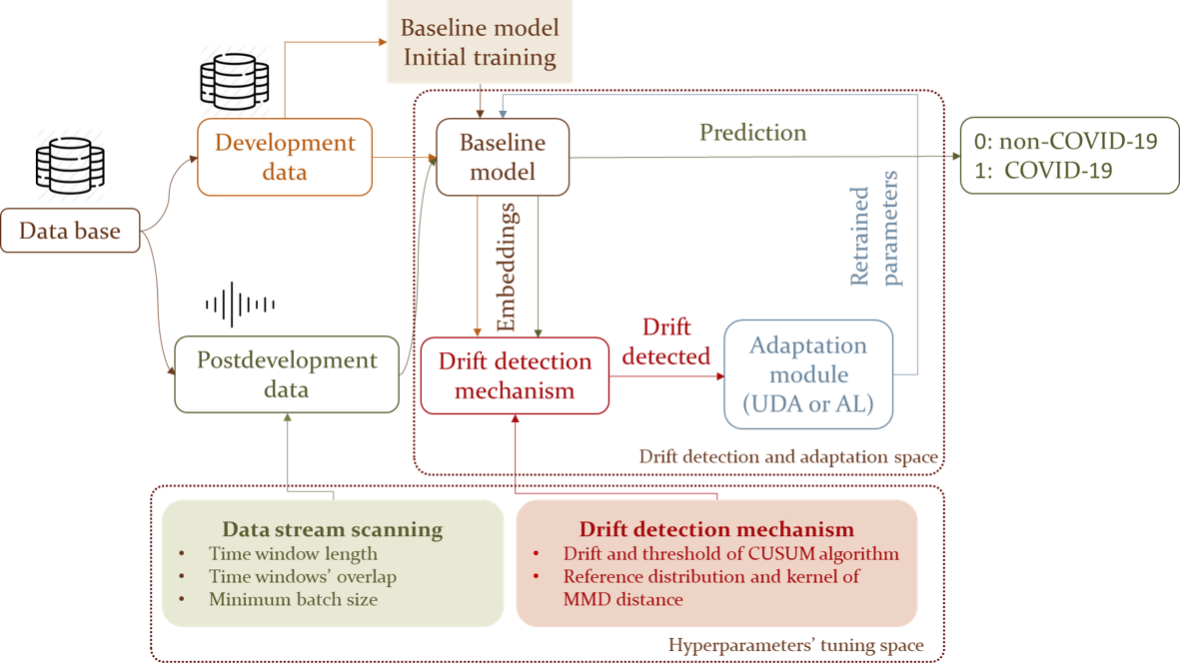
Overview of the proposed framework. The data are split into a labeled development set for training the baseline model and an unlabeled postdevelopment set for evaluation. The framework includes three modules: (1) a baseline model trained for binary classification, (2) a drift detection mechanism that monitors model performance in postdevelopment data, and (3) an adaptation module that retrains the model using unsupervised domain adaptation (UDA) or active learning (AL) when drift is detected. CUSUM: cumulative sum; MMD: maximum mean discrepancy.

The abstract architecture of the proposed framework is depicted in Figure 1. It comprises 3 distinct modules, combining a deep neural network with a drift detection mechanism and appropriate adaptation modules with the aim of addressing differences between data distributions of the development and postdevelopment periods. These modules are explained subsequently.

The first module, called a baseline model, is based on a convolutional neural network, which processes input data instances and estimates the probability of COVID-19 presence.

The second module is the drift detection mechanism that is responsible for the identification of drifts in the data, implying changes in COVID-19 detection patterns. It monitors the performance of the baseline model through the detection of significant discrepancies between the development data and the postdevelopment data. To this end, a modified version of the cumulative sum (CUSUM) algorithm is used, with the MMD distance being used to measure the distance between data distributions from the development and postdevelopment periods. A set of hyperparameters, which are appropriately adjusted, is included in the CUSUM algorithm (ie, drift and threshold) and the MMD distance (ie, reference distribution and kernel).

The third module is the adaptation module. It facilitates the adaptation process within the system. It enables the model to dynamically adjust and learn from postdevelopment data, ensuring continuous improvement and robustness against evolving COVID-19 characteristics. Two different approaches using divergence-based UDA and AL were investigated for harnessing postdevelopment, unlabeled data for model retraining, with the aim of enhancing the performance and improving the generalization abilities of the baseline model.

The proposed framework’s operation is based on the adoption of a batch-based approach for the processing of data instances and the application of a fixed time window (with parametrized overlap between successive windows) to monitor the data stream for changes. The time window length, overlap between successive time windows, and minimum batch size, along with the hyperparameters of the drift detection mechanism, are appropriately validated to ensure optimal performance for each dataset.

**Figure 2:**
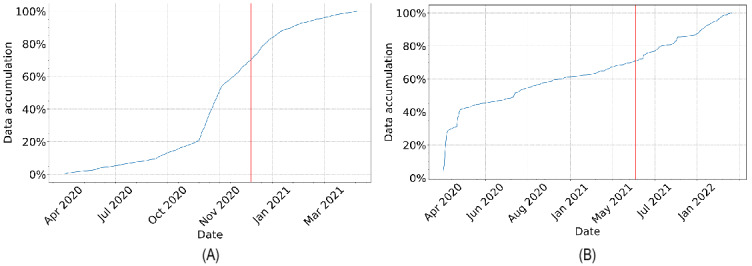
COVID-19 Sounds (A) and Coswara (B) data streams over time. A 70:30 partition of the data into development and postdevelopment sets is applied, marked by the red line. The development set was divided into training, validation, and test subsets (60:20:20). Care was taken to avoid participant overlap across all subsets and between development and postdevelopment periods.

The development and evaluation of the proposed framework were based on the use of cough recordings from the COVID-19 Sounds [44] and Coswara [55] datasets. Both datasets were partitioned into development and postdevelopment sets based on chronological order. Figure 2 illustrates the partition of data, while Table 1 summarizes the distribution of COVID-19 positive samples for both datasets.

#### Baseline Model

**Figure 3:**
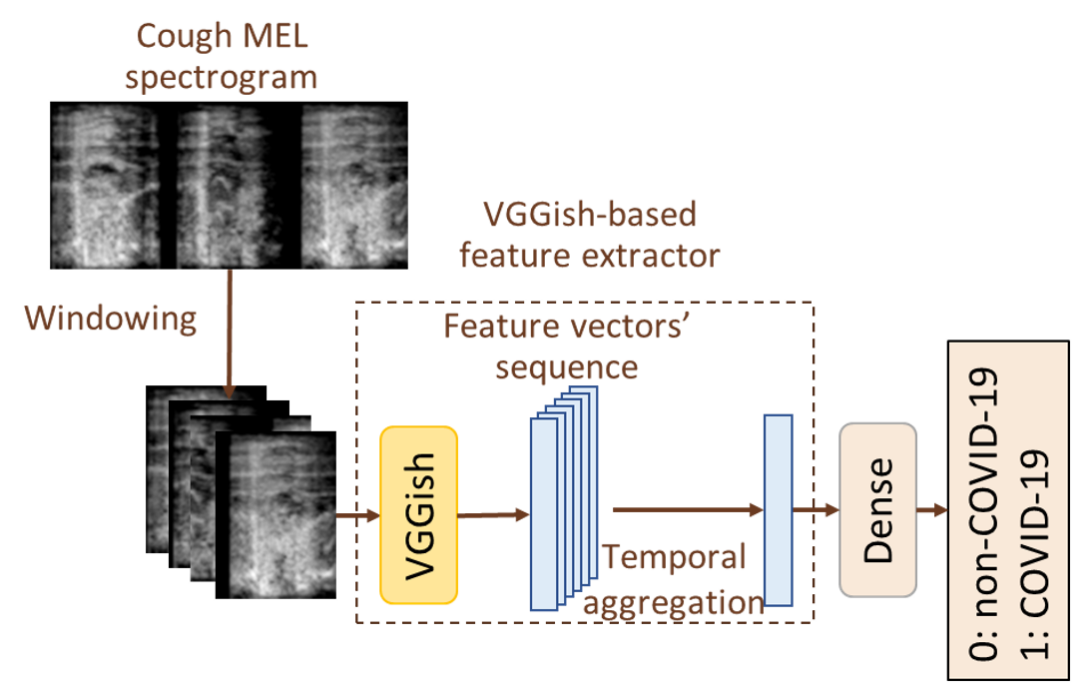
Baseline model architecture.

The baseline model of the proposed framework was built upon the widely used VGGish pretrained model [56,57], which was selected due to its remarkable performance on audio classification tasks [16,58]. The VGGish model is a deep convolutional neural network model trained on a large-scale audio dataset to learn hierarchical representations of audio signals. In the framework of this study, the VGGish model was used to extract discriminative features from segments of mel spectrograms with the aim of capturing relevant acoustic patterns and distinguishing COVID-19 coughs sounds from non–COVID-19 coughs sounds. Figure 3 shows the general architecture of the baseline model used.

To adapt the VGGish model to the specific task of this study, a time-distributed approach was used. To this end, the VGGish feature extractor was applied on each segment of the mel spectrogram, resulting in a sequence of feature vectors that represented the temporal evolution of acoustic characteristics within the cough signal. In order to summarize the temporal dynamics captured by the model, the mean value for each feature across the entire sequence was calculated. Following the temporal aggregation, a dense layer with a single node based on the nonlinear sigmoid activation function was used to process the aggregated feature vectors and calculate the final output of the model.

In order to address the imbalanced nature of the datasets, the binary focal cross-entropy loss function was used for training the baseline model due to its ability to focus on rare examples [59]. This loss function effectively assigned higher weights to misclassified samples, thereby alleviating the impact of class imbalance and improving overall performance. For optimization, the adaptive moment estimation optimizer was used due to its efficient and adaptive nature [60]. A learning rate equal to 10^–4^ was used, while the exponential decay rate for the first and second moment estimates was 0.9 and 0.999, respectively.

During training, the labeled samples of the development set were considered to minimize the chosen loss function. A batch size of 32 and 100 epochs was used, which is a commonly used default training scheme used in multiple studies [61,62]. The validation score was used for monitoring the model’s convergence, and an early stopping regularization technique was applied. After convergence, the performance of the trained model was evaluated on a test subset sampled from the development period and on the entire postdevelopment set.

#### Drift Detection Mechanism

##### Overview

**Figure 4:**
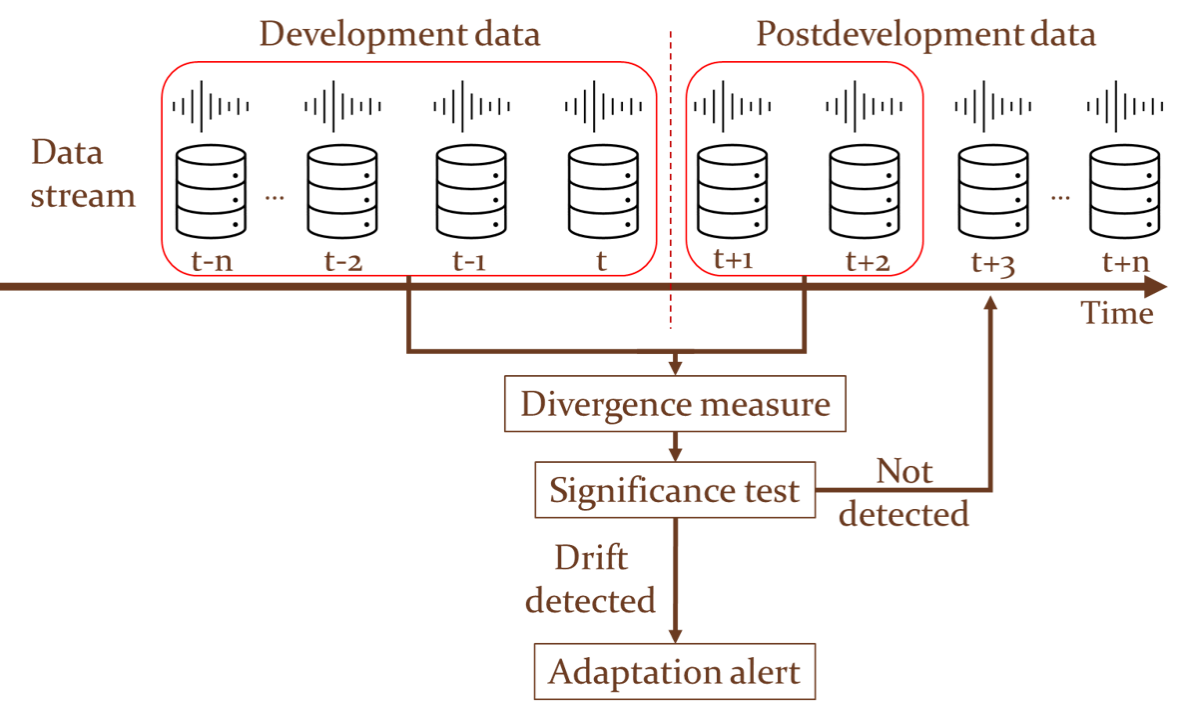
Proposed drift detection mechanism. Data are processed in chronological order based on their acquisition time point (t).

The proposed drift detection mechanism entailed divergence monitoring using the MMD distance and the implementation of the CUSUM algorithm [63,64] for generating drift alerts. The data were used in a chronological order to monitor the performance of the model. A batch-based approach was adopted for monitoring and processing data instances. An overview of the proposed drift detection mechanism is provided in Figure 4.

##### Monitoring Divergence With MMD Distance

To effectively track the dissimilarity between the development and postdevelopment data, the MMD distance was adopted, which was computed by comparing the corresponding embeddings extracted by the VGGish feature extractor, as described in the earlier subsection. These embeddings served as a representation of the data distribution and were used for calculating the MMD distances between batches of the postdevelopment and the development data, with the embeddings of the latter constituting the reference distribution. The MMD distance value between 2 batches of data is given by the following equation:



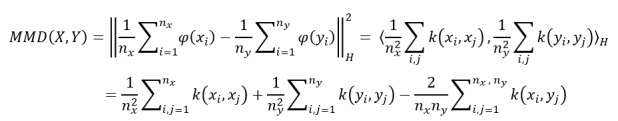



where *X* represents the distribution of the embeddings of the development data *x_i_* (reference distribution), with *n_x_* embeddings in total; *Y* represents the distribution of the embeddings of postdevelopment data *y_i_*, with *n_y_* embeddings in total; *φ*(.) represents the feature mapping function used to transform the embeddings into a high-dimensional space (VGGish model); *k*(.,.) is a kernel function that computes the similarity between two inputs; and〈.,.〉*_H_* denotes the inner product in the Hilbert space induced by the kernel function.

In this study, the use of 3 different kernels (linear, polynomial of degree 2, and Gaussian) was investigated.

##### CUSUM Algorithm

After calculating the divergence between the development and postdevelopment data, an implementation of the CUSUM algorithm was deployed for detecting points of significant increase in the divergence measure. CUSUM is a change detection algorithm that is widely used to identify drifts or changes in time series data [65-67], particularly when the exact nature of the change is unknown or when there is a need to continuously monitor data for detecting changes. CUSUM is widely adopted for real-time monitoring and surveillance applications in various fields, including quality control, signal processing, and anomaly detection.

In this study, the CUSUM algorithm was tailored to match the specific characteristics of the deep learning model and the monitored MMD distance. The proposed implementation introduced the calculation of relative differences between successive values instead of their corresponding absolute values, thus enabling the original CUSUM algorithm to effectively align with the behavior of the MMD distance and the desired level of sensitivity to changes. Therefore, the drift and threshold values represented the tolerance range of relative change in successive values and the minimum cumulative relative change required to trigger a change detection event, respectively.

#### Adaptation Mechanism

##### Overview

Upon the triggering of an alert by the drift detection mechanism, an adaptation mechanism based on model retraining was activated to update the baseline model. The proposed adaptation mechanism aimed at enhancing the performance and improving the generalization abilities of the baseline model. Two different approaches based on UDA and AL were explored for the development of the adaptation mechanism.

##### UDA Approach

The UDA approach involved feeding the model with a batch of postdevelopment data samples, along with a batch of samples from the development set. The model was then trained jointly on two tasks: (1) correctly classifying the labeled development data and (2) minimizing the MMD distance between the embeddings of the development and postdevelopment batches. In this way, the model was trained to solve task 1 using domain-invariant features (development and postdevelopment data), aiming at the minimization of 2 loss functions. The first loss function considered the model’s output on samples of the development dataset, essentially using supervised learning. The second loss function was based on the divergence between the distributions of the postdevelopment data and development data batches using the MMD distance. The Gaussian kernel is represented as follows:



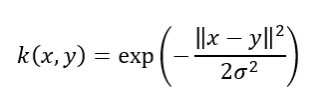



It was selected to be used for the MMD distance calculation due to its ability to distinguish between distributions with differences in any order of moments [36,68], as demonstrated by its Maclaurin series representation as follows:



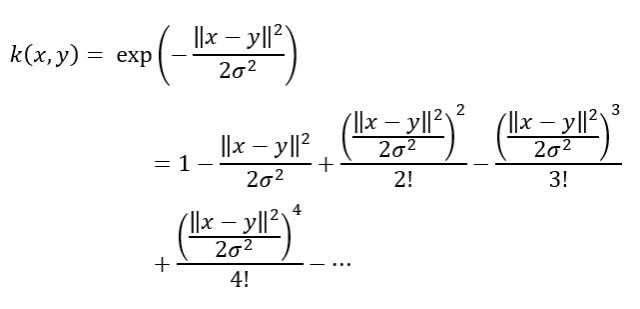



In contrast, the linear kernel cannot distinguish between distributions with the same mean but different higher-order moments, while the polynomial kernel of degree 2 is unable to differentiate between distributions that have the same mean and variance but differ in higher-order moments.

**Figure 5:**
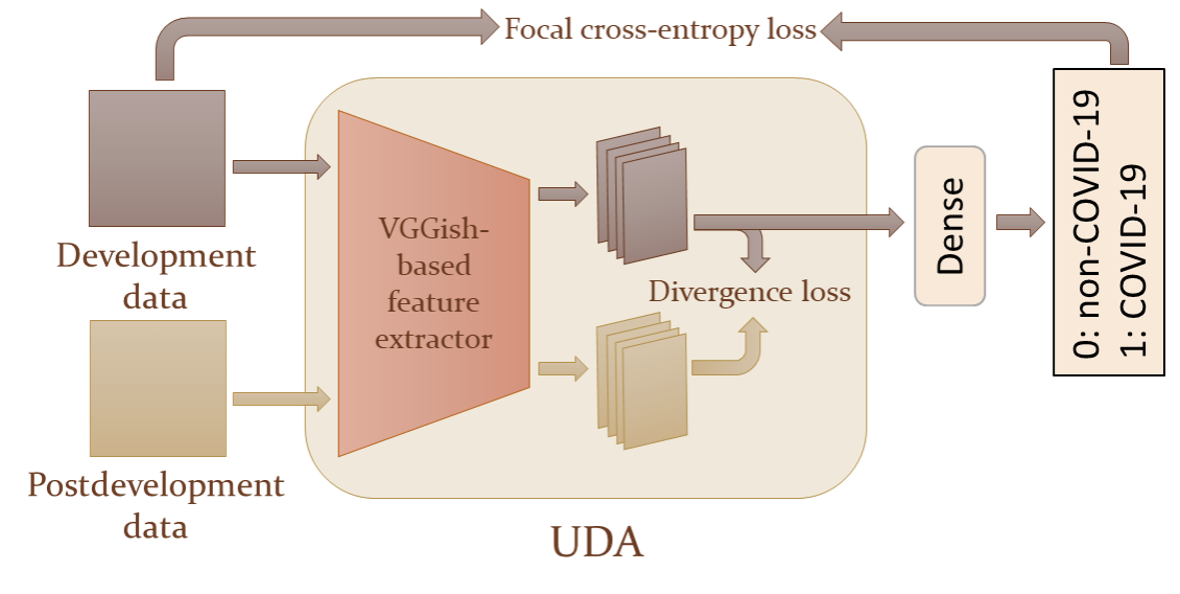
Unsupervised domain adaptation (UDA) process. The model was fed with a batch of postdevelopment data samples, along with a batch of samples from the development set and was then trained jointly (1) to correctly classify the labeled development data and (2) to minimize the maximum mean discrepancy distance between the embeddings of the development and postdevelopment batches.

During retraining, both loss functions were minimized simultaneously to enhance the model’s adaptability to the postdevelopment data while preserving its previous knowledge. Figure 5 shows an overview of the UDA method.

##### AL Approach

The second adaptation approach incorporated AL principles into the retraining process. Building upon the drift detection mechanism, a methodology was developed that was able to identify informative data points, incorporating both diversity and uncertainty estimation [41,42]. Once a period of divergence was detected by the drift detection mechanism, uncertain instances were selected from the divergent batch of data. To achieve this, the *z* scores of the model’s outputs on the divergent data were calculated, and the data samples whose output fell within 1 SD around the mean value of the model’s predictions were defined as uncertain. Samples within this uncertainty range were selected, thus prioritizing the inclusion of challenging and informative instances during retraining, with the ultimate goal of enhancing the model’s generalization capabilities.

Considering that this adaptation method involved selecting informative unlabeled data and using them as labeled data, it was essential to compare the results of AL with those obtained through random sampling. The number of randomly sampled data samples was equal to the number of data points used in the adaptation phases of the AL approach.

### Ethical Considerations

No ethics approval was required for this study as it was not human participant research and did not include experiments on humans and the use of human tissue samples. As indicated in the data availability statement, the development of the presented methods was based on the use of a publicly available dataset and a dataset which was granted by a third party following the submission of relevant request. The Coswara data collection procedure was approved by the Institutional Human Ethics Committee, at the Indian Institute of Science, Bangalore. The informed consent was obtained from all participants who uploaded their data records. All the data collected was anonymized, and excluded any participant identity information. The COVID-19 sounds study was approved by the ethics committee of the Department of Computer Science at the University of Cambridge. Informed consent was given by the users through the mobile app.

## Results

### Baseline Model

**Figure 6:**
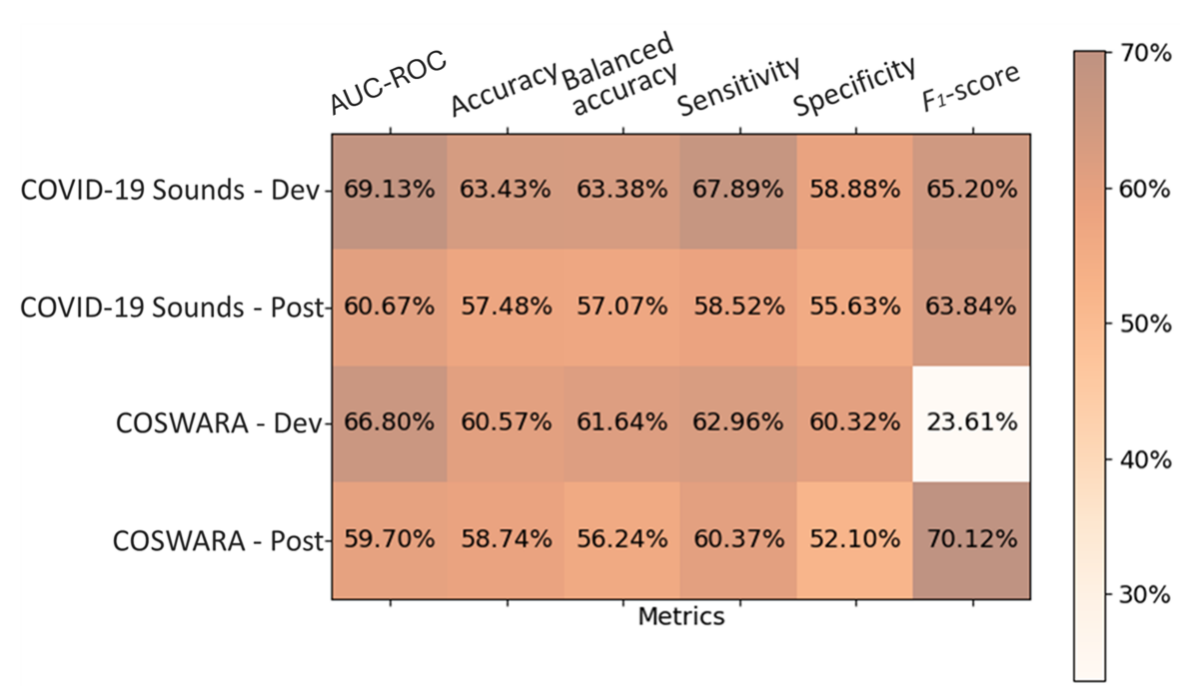
Performance evaluation of the baseline model on the development and postdevelopment data from the COVID-19 Sounds and Coswara datasets. The model’s performance was assessed using the area under the receiver operating characteristic curve (AUC-ROC), accuracy, balanced accuracy, sensitivity, specificity, and F1-score. The test subset from development data is referenced as Dev, while Post refers to the entire postdevelopment period.

The baseline model was assessed in terms of its ability to accurately detect COVID-19 cases in the presence of variations or shifts in the data. Particularly, the performance evaluation on the 2 datasets considered for the development and postdevelopment periods is reported in Figure 6.

On the basis of the results obtained for the COVID-19 Sounds dataset, it was observed that the baseline model achieved superior performance on the test subset of the development period in terms of the area under the receiver operating characteristic curve (AUC-ROC; 69.13%) and sensitivity (67.89%) compared to the best model performance reported in the literature [16] (AUC-ROC: 66%; sensitivity: 59%; specificity: 66%), despite considering a smaller amount of labeled data for training and validation (619 vs 1062 instances). The baseline model achieved a satisfactory *F*_1_-score (65.2%) but demonstrated quite low specificity, correctly classifying 58.9% (63/107) of instances from the negative class. The performance of the baseline model on the postdevelopment data demonstrated a significant decline in the AUC-ROC, the *F*_1_-score, sensitivity, and specificity, as reported in Figure 6.

In the case of the Coswara dataset, the baseline model displayed moderate discriminative ability on the development data, achieving an AUC-ROC value of 66.8%, while the accuracy, sensitivity, and specificity scores were 60.57%, 62.96%, and 60.32%, respectively. The highly imbalanced distribution of the 2 classes in the development data (165/1395, 11.82% positive vs 1230/1395, 88.18% negative) posed a significant challenge for the model, as highlighted by the notably low *F*_1_-score, a metric that exclusively focuses on positive instances. The model’s discriminative power on the postdevelopment data presented a decline, as indicated by the AUC-ROC, specificity, and sensitivity (Figure 6). The high value obtained for the *F*_1_-score metric was related to the presence of class imbalance with reversed minority (negative) and majority (positive) classes in the postdevelopment data with respect to the class distribution of the development data, which led to a misleading perception that the model’s performance had significantly improved. A more thorough analysis of this issue is provided in the Discussion section.

### Hyperparameters’ Tuning

The hyperparameters of the drift detection mechanism were fine-tuned across both datasets to maximize detection accuracy. For the COVID-19 Sounds dataset, the best-performing configuration incorporated a 7-day window, 3-day overlap, a minimum batch size of 40 samples, a polynomial kernel for the MMD calculation, and CUSUM drift and threshold values of 0.2 and 0.5, respectively, and achieved an accuracy score of 91.3%, sensitivity of 88.5%, and specificity of 93.2%. For the Coswara dataset, the optimal setup included a 10-day window, no overlap, a minimum batch size of 20 samples, and CUSUM drift and threshold values of 0.2 and 0.7, respectively, leading to an accuracy score of 89.7%, sensitivity of 85.2%, and specificity of 92.1%. Further details on the tuning procedure and hyperparameter selection are provided in Multimedia Appendix 1.

### UDA Approach

**Figure 7:**
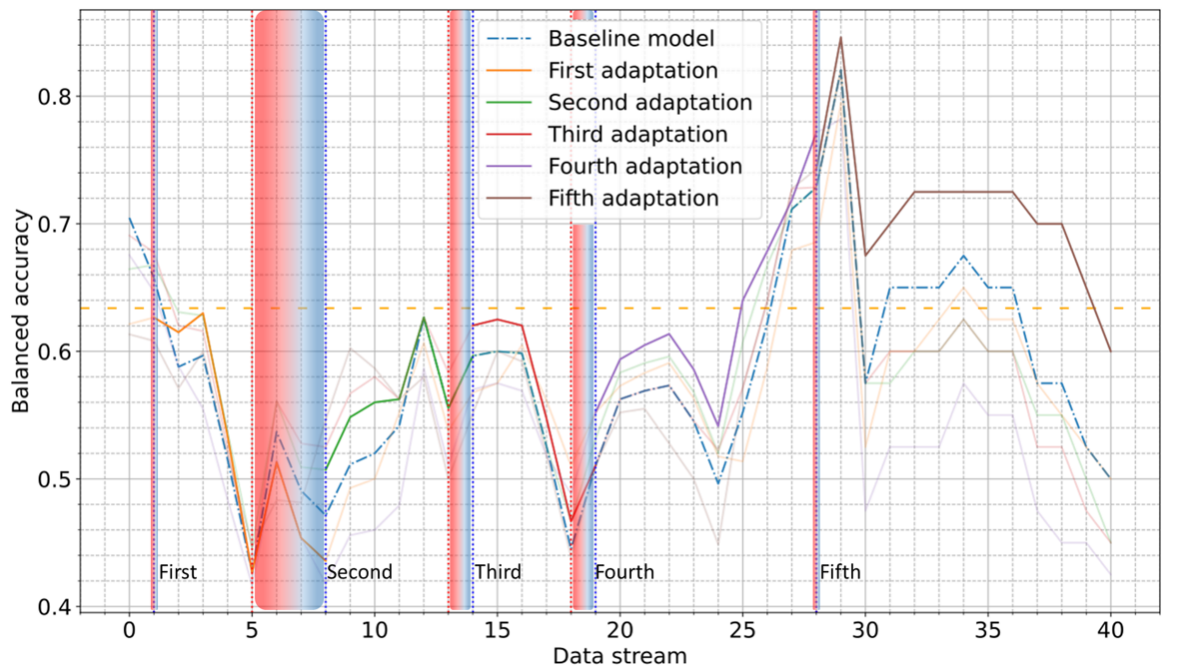
The obtained balanced accuracy score across the data batches of the entire postdevelopment period of the COVID-19 Sounds dataset using unsupervised domain adaptation. The orange dashed line is used to indicate the performance on the test subset of the development period (benchmark). Vertical red and blue dotted lines indicate the start and end of each alert period.

A comparative assessment of the model’s performance, before and after each UDA adaptation phase, was carried out, with balanced accuracy on the test subset of the development period serving as a benchmark. For the COVID-19 Sounds dataset, considerable improvement was achieved after each adaptation phase, particularly following the fourth (up to 15%) and fifth (up to 24%) adaptations (Figure 7). The model consistently outperformed the baseline model, demonstrating the effectiveness of the proposed approach in mitigating concept drift. It is noteworthy that by correctly identifying periods of drift, the drift detection mechanism efficiently prevented the degradation of the model’s performance in a timely manner while also contributing to sustaining the model’s performance closer to the development period benchmark.

**Figure 8:**
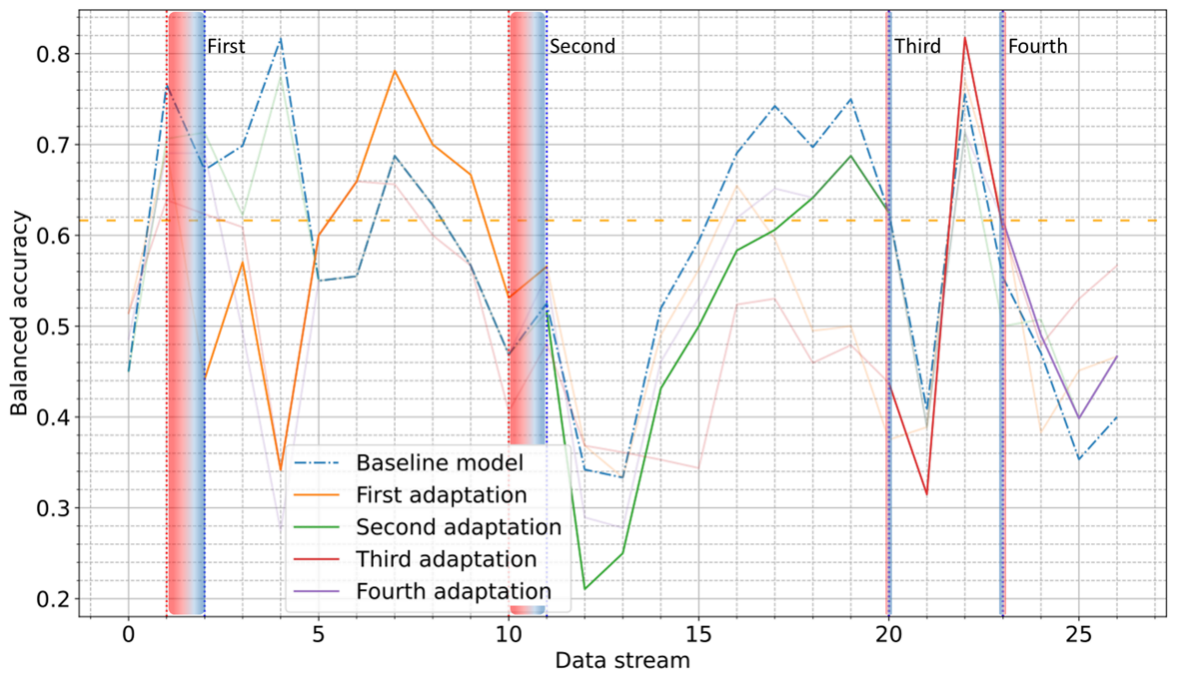
Balanced accuracy score through the entire postdevelopment period on Coswara dataset using unsupervised domain adaptation. The orange dashed line is used to indicate the performance on the test subset of the development period. Vertical red and blue dotted lines indicate the start and end of each alert period.

The results obtained for the Coswara dataset are depicted in Figure 8. It was observed that while some adaptations (eg, fourth) exhibited up to 15% improvement, others initially led to a significant yet short-lasting decline in the model’s performance, before ultimately demonstrating the ability to recover. Despite these fluctuations, the drift detection mechanism effectively generated timely alerts, preventing prolonged performance degradation.

A detailed study of each adaptation’s impact on various performance metrics (AUC-ROC, accuracy, balanced accuracy, sensitivity, specificity, and *F*_1_-score) for both datasets was also carried out. The results of the COVID-19 Sounds dataset showed that the UDA approach significantly enhanced the model’s performance in terms of all evaluation metrics. However, the application of the UDA approach on the Coswara dataset produced less consistent results. Overall, the performed adaptations exhibited varying effects on the model’s performance across the considered evaluation metrics. The complete results of this analysis are presented in Multimedia Appendix 2.

### AL Approach

**Figure 9:**
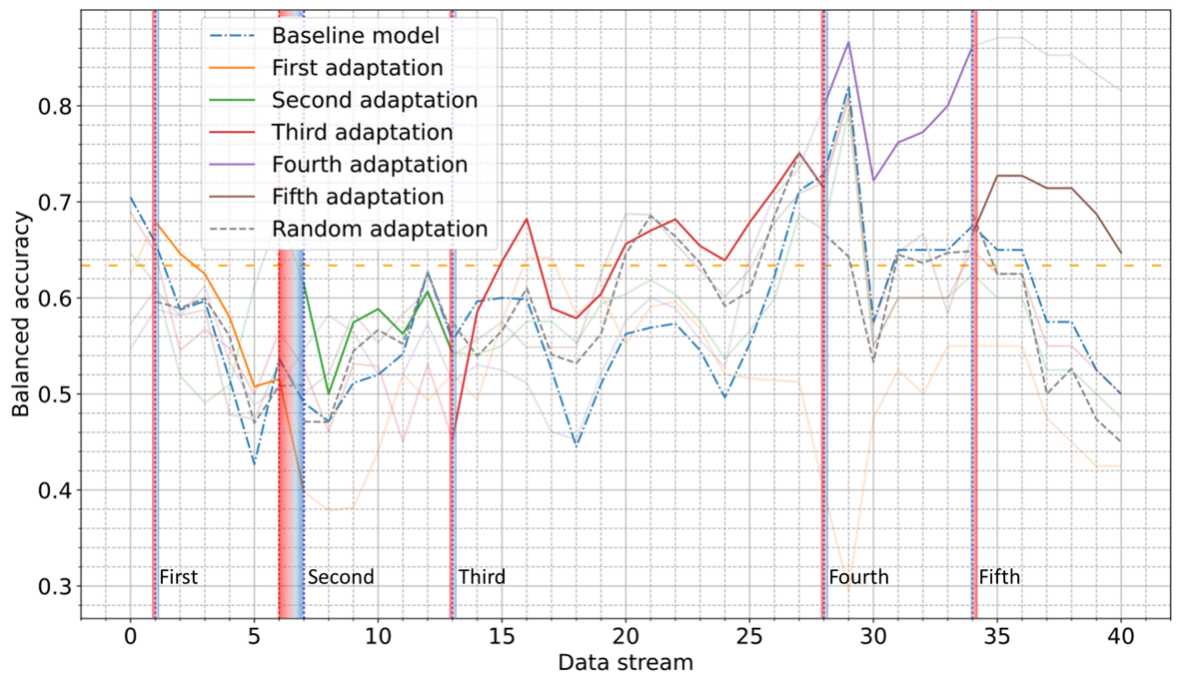
Balanced accuracy score through the entire postdevelopment period on the COVID-19 Sounds dataset using active learning. The orange dashed line indicates the performance on the test subset of the development period. Vertical red and blue dotted lines indicate the start and end of each alert period.

The proposed AL approach was evaluated by comparing the performance of the model after each AL-based retraining phase with that of the baseline model, as well as the model following retraining, using random sampling. Considering the COVID-19 Sounds dataset, Figure 9 demonstrates the observed balanced accuracy score across the entire data stream, indicating a substantial and lasting improvement with respect to the baseline model following each adaptation. Overall, each AL-driven adaptation significantly enhanced balanced accuracy, with the third adaptation improving performance by up to 30% over a broad period of 15 batches, and the fourth adaptation surpassing a 95% balanced accuracy score while achieving an improvement of up to 25% compared to the baseline model. The superiority of the proposed AL approach over random sampling was evident across 90% (36/40) of the data batches.

**Figure 10:**
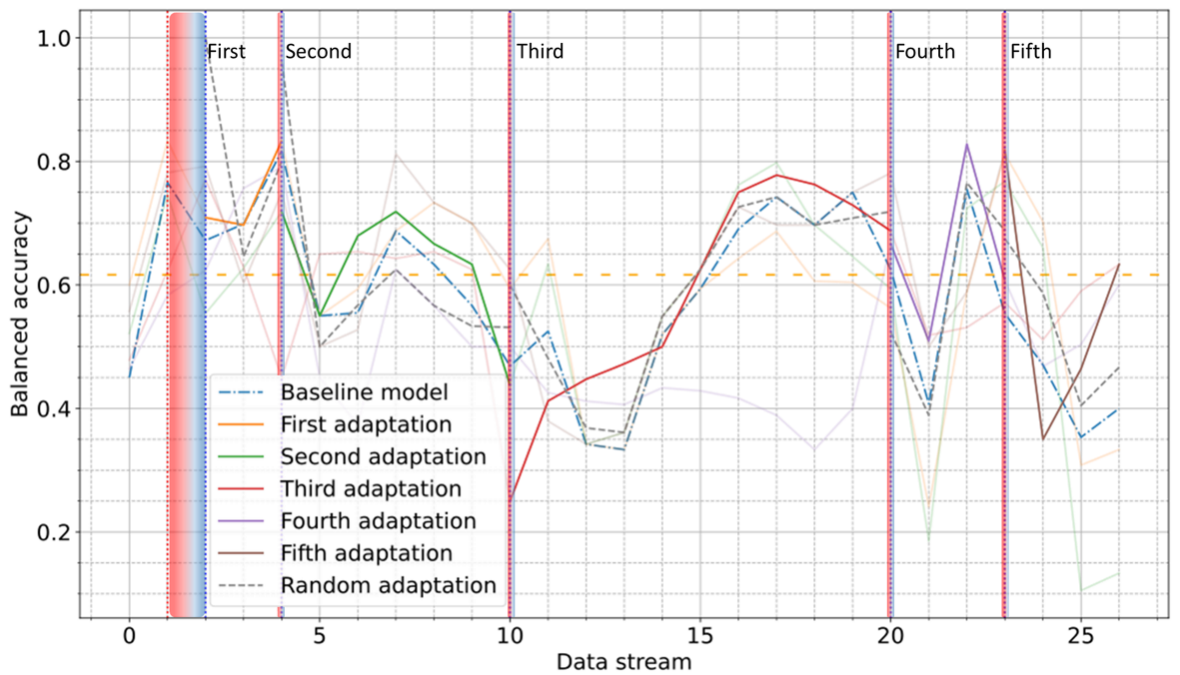
Balanced accuracy score through the entire postdevelopment period on the Coswara dataset using active learning. The orange dashed line indicates the performance on the test subset of the development period. Vertical red and blue dotted lines indicate the start and the end of each alert period.

In the case of the Coswara dataset, Figure 10 shows that model adaptations led to improved performance during most postalert periods, particularly after the third (up to 40%) and fifth (up to 60%) adaptations. Occasional fluctuations of limited duration were observed, mostly in the early batches of the postalert periods, where the baseline model outperformed the AL-based model. As compared to random sampling, AL remained superior in most cases, further confirming its effectiveness in selecting informative data.

Further insights were derived through per-metric performance comparisons, including AUC-ROC, accuracy, sensitivity, specificity, and *F*_1_-score, across adaptations for the 2 studied datasets. Most AL-based adaptations for the COVID-19 Sounds dataset yielded improved performance in all the considered evaluation metrics with respect to the baseline model and the random sampling approach. In the case of the Coswara dataset, the AL approach led to an overall improvement in the model’s performance over the baseline model and the random sampling approach, except for certain metrics and adaptations. The results obtained are presented in detail in Multimedia Appendix 2.

## Discussion

### Principal Findings

This study proposed a drift-adaptive framework for COVID-19 detection using crowdsourced cough audio recordings and evaluated its effectiveness in addressing temporal data drifts, confirming its ability to sustain model performance over time.

The proposed framework addressed challenges imposed by dynamic, nonstationary environments caused by a pandemic by incorporating a drift detection mechanism and appropriate adaptation strategies. The proposed approach focused on the temporal evolution of data distributions in a real-world scenario, unlike previous studies that did not explicitly consider temporal aspects [15,16,46-50]. The evaluation of the introduced framework provided evidence regarding its ability to maintain model performance, thus highlighting its potential to facilitate the identification of new cases in the evolving context of a pandemic.

A baseline model that was able to detect COVID-19 positive cases using cough recordings was trained and evaluated. During the development period, the model achieved an AUC-ROC of 69.1% and 66.8% on the COVID-19 Sounds and Coswara datasets, respectively. However, in the postdevelopment period, there was a notable decline in the baseline model’s performance, reflected in an AUC-ROC of 60.7% and 59.7%, respectively, thus suggesting the potential presence of concept drift. These findings motivated the development of the proposed approach, which leveraged robust drift detection and efficient adaptation mechanisms to maintain the model’s performance in the face of evolving data distributions.

The results obtained indicated the efficacy of the proposed drift detection mechanism and provided evidence regarding its ability to enhance the robustness and adaptability of deep learning models in dynamic environments. The combination of the MMD distance monitoring and the use of the CUSUM algorithm for adaptive detection of abrupt changes, which reflect a growing divergence between the reference distribution (development data) and the postdevelopment data, enabled the timely and robust detection of performance degradation. The use of the CUSUM algorithm, tailored to the characteristics of each dataset, ensured the generation of accurate alerts for significant changes in the monitored MMD distance, thus minimizing false alerts and preventing unnecessary interventions.

Two distinct retraining strategies based on UDA and AL were used to mitigate performance degradation in this study. Notably, the use of UDA in this study focused on achieving continuous adaptation in the presence of real-world data drift, unlike previous studies [37] where UDA was used to address static cross-dataset generalization. Similarly, this study incorporated AL as an adaptation mechanism, ensuring continuous model refinement in response to data distribution shifts, in contrast to previous studies [40] where AL was used for reducing annotation costs by selecting the most informative samples for initial model training.

Regarding the use of UDA, the results obtained from the analysis of the COVID-19 Sounds dataset showed significant improvement in the model’s discriminative ability. The comprehensive examination of the adaptation phases based on multiple evaluation metrics mostly revealed improvements in the balanced accuracy with respect to the baseline model’s performance, ranging from 10% to 20%.

The aforementioned findings align with UDA’s core advantages, which include cost-effectiveness and adaptability to dynamic environments through the model’s adaptation to the target domain’s data distribution without requiring labeled target domain samples. This approach is particularly valuable when labeled data from the target domain are scarce or expensive to obtain, as is often the case in emerging pandemic scenarios.

The application of the UDA approach on the Coswara dataset yielded less consistent results. The overall comparison between the adapted models and the baseline model revealed that the adaptation had diverse effects on the model’s performance in terms of the evaluation metrics considered.

**Figure 11:**
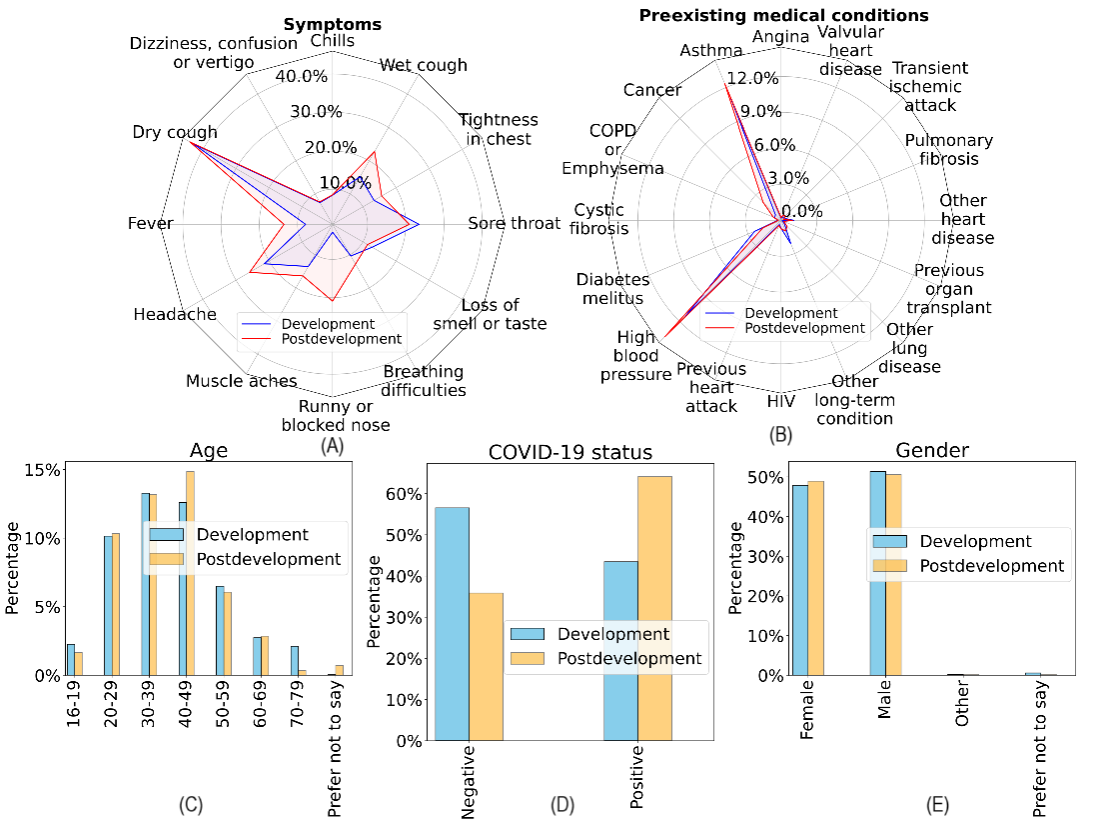
Descriptive statistics for COVID-19 Sounds development and postdevelopment data reveal moderate changes. The disease exhibited moderate shifts in both its prevalence and the frequency of related symptoms. The 2 data subsets shared similar characteristics in terms of age, gender, and medical history of individuals. COPD: chronic obstructive pulmonary disease.

**Figure 12:**
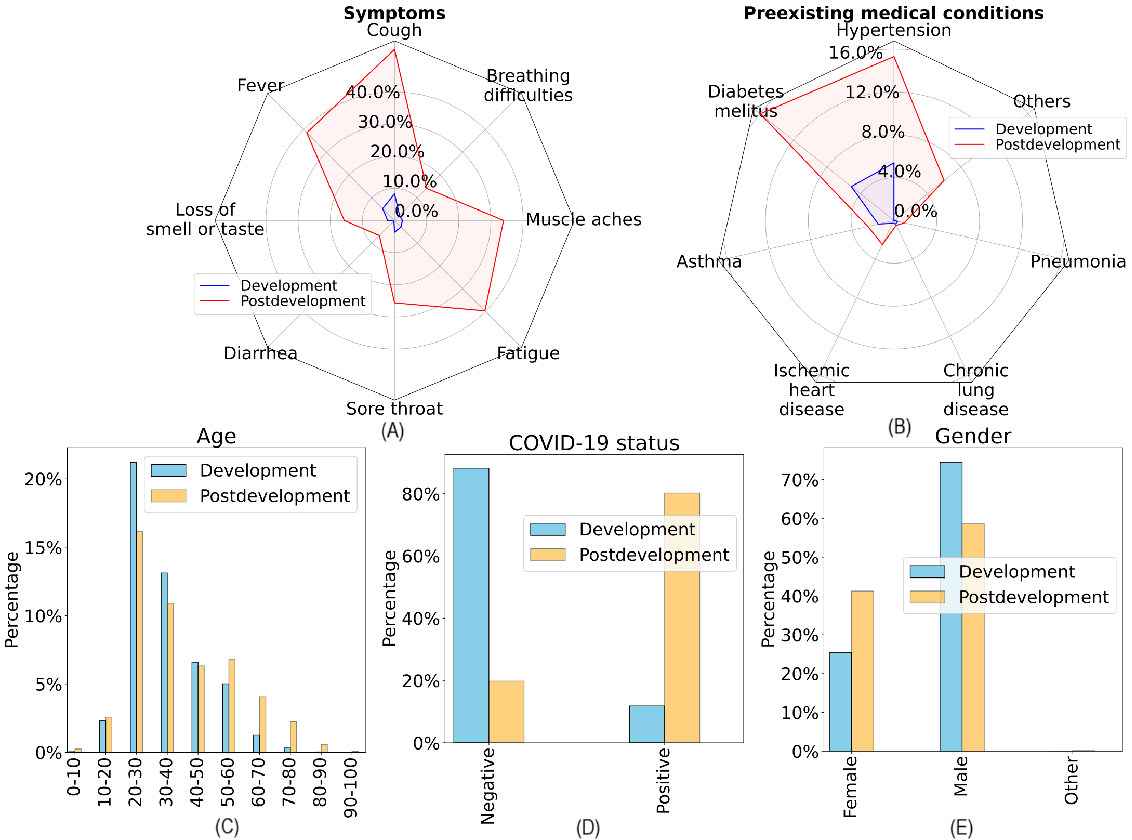
Descriptive statistics for Coswara development and postdevelopment data reveal profound differences in demographic characteristics, symptoms, and preexisting medical conditions between the development and postdevelopment periods. The representation of positive and negative classes in the development data is reversed in the postdevelopment data.

The difference in the effectiveness of the UDA approach on the COVID-19 Sounds and Coswara datasets may be attributed to differences in the datasets’ characteristics. Figures 11 and 12 illustrate selected descriptive statistics of the development and postdevelopment data of the COVID-19 Sounds and Coswara datasets. As per Han et al [16], the COVID-19 Sounds dataset used in this study had undergone meticulous curation to eliminate biases as a result of methodological decisions, thus enabling the development of unbiased models. In the case of the Coswara dataset, significant differences were observed in terms of COVID-19 prevalence and the frequency of related symptoms between the development and postdevelopment data, which may be attributed to the presence of age and gender biases [69]. In this study, handling the data in chronological order implicated different levels of data biases present across the various adaptation periods, which may arise in emerging pandemic scenarios.

The AL approach resulted in a more prominent improvement in the models’ performance compared to UDA. This underscored the power of actively selecting informative samples for labeling, which aids in refining the model’s understanding of the target domain. Thus, by optimizing both adaptation to the target data and the use of labeling resources, AL is considered promising for ensuring model performance in data-scarce scenarios, such as during a pandemic.

Given that both UDA and AL achieved varying levels of performance improvement on the COVID-19 Sounds and Coswara datasets, it is essential to consider their limitations and potential challenges. UDA relies on the assumption that the source and target domains share some underlying similarity. In the presence of significant differences, adaptation might not yield substantial improvements. By contrast, AL’s performance is determined by human labeling expertise, which is associated with the rise of the related costs and depends on the reliability of the existing diagnostic tests. If the chosen samples are mislabeled, the model’s performance could suffer. Moreover, AL’s performance is sensitive to the selection of labeled samples, which might introduce biases.

**Figure 13:**
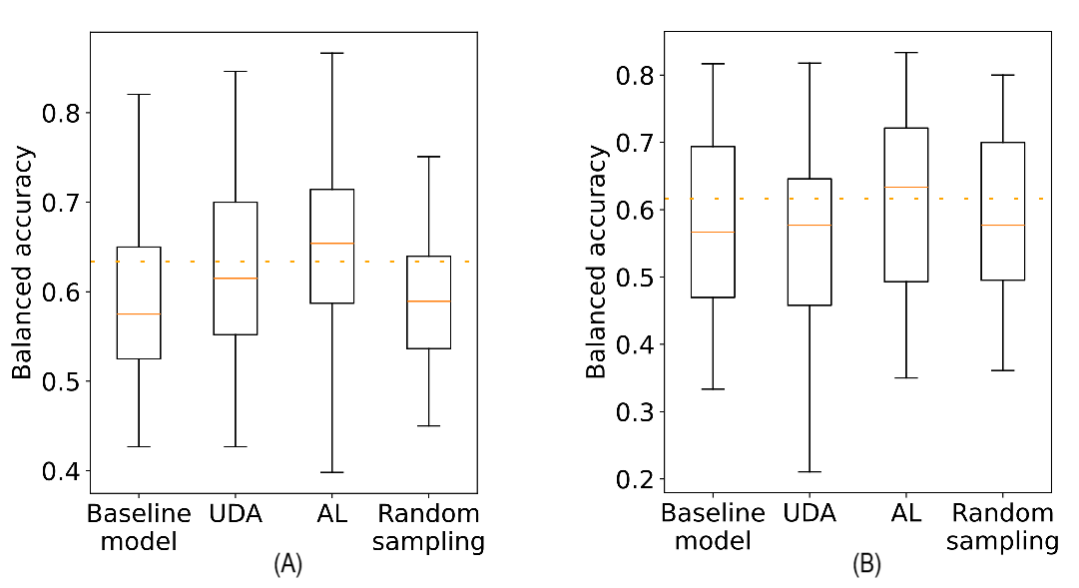
Box plots of the balanced accuracy scores across the entire postdevelopment period using the baseline model, the unsupervised domain adaptation (UDA) approach, the active learning (AL) approach, and the random sampling approach for the COVID-19 Sounds (A) and Coswara (B) datasets. The orange dashed line indicates the performance of the baseline model on the test subset of the development period (benchmark).

The aforementioned observations suggest that the proposed adaptation mechanisms effectively addressed the individual challenges linked to the special characteristics of each dataset and mitigated the effects of concept drift during critical periods corresponding to batches in proximity to the alert periods. Figure 13 summarizes the model’s performance obtained by applying each adaptation approach on the postdevelopment period of the 2 datasets and shows that both approaches succeeded in maintaining the models’ performance closer to the development period’s benchmark.

Further evidence regarding the proposed framework’s ability to detect concept drift and maintain model performance was provided through a detailed analysis of stratified heterogeneity (SH) using the Q statistic [70-73]. The findings obtained indicated that COVID-19 presence was stratified across time, age, and gender, with a strong correlation to performance fluctuations. Notably, detected input drift alerts aligned with statistically significant differences of SH across strata (*P* values for all pairwise comparisons are reported in Multimedia Appendix 3), indicating the presence of drifts in the ground truth label, which provided further evidence on the effectiveness of the proposed drift detection mechanism. Moreover, the superiority of the UDA- and AL-based adapted models’ performance over that of the baseline model and the adapted model using random sampling was observed for most batches, presenting statistically significant differences in SH. The detailed SH analysis that was conducted is provided in Multimedia Appendix 3.

These results highlight the importance of combining effective drift detection mechanisms and intelligent adaptation modules in addressing concept drift. Together, these components form a robust framework that enables the model to continuously adapt to changing data conditions, thereby maintaining its discriminative power and overall performance over time.

### Limitations

Certain potential limitations of the proposed study should be acknowledged. First, although the proposed framework successfully detects and responds to performance degradation based on the MMD distance, it does not explicitly interpret the underlying causes of the drift. Understanding the sources of the drift could enhance trust in the detected drifts and enable efficient targeted interventions toward sustaining model performance. Moreover, by design, this study isolates and investigates temporal drift as the primary source of distributional change. While this focused approach allows for detailed analysis, it limits exploration of other drift types, such as cross-dataset drifts or interdemographic variability, which may arise in broader deployment scenarios. Finally, the framework was applied for COVID-19 detection from cough data; the integration of multimodal inputs, including breathing and voice data, is straightforward and could further improve the model’s performance.

### Conclusions

The significance of the proposed approach lies in its reliance on data-efficient techniques. By minimizing the dependence on labeled data, the proposed framework enables the accurate detection of COVID-19 cases even in the absence of comprehensive labeling resources. This aspect becomes particularly crucial when considering the value of a deep learning–based detection model during the early stages of a new pandemic or when dealing with emerging viral variants that may not be adequately detected by existing diagnostic tools. Thus, the proposed framework is able to contribute toward a more generalizable approach that can be applied to future pandemics or novel variants. By collecting knowledge and formulating a well-defined framework, a basis for rapid adaptation and deployment of disease detection tools is established, ensuring timely and accurate identification of infectious diseases.

## Data Availability

The COVID-19 Sounds dataset analyzed during this study is not publicly available due to the requirement of license acquisition by the Department of Computer Science at Cambridge University, but is available to academic institutions for academic research purposes upon the submission of a relevant request to the mobile systems group and the signing of a data transfer agreement. Please contact covid-19-sounds@cl.cam.ac.uk to obtain it. The Coswara dataset analyzed during this study is available in the GitHub repository [74].

## Appendix

Multimedia Appendix 1 Hyperparameters’ tuning.

Multimedia Appendix 2 Assessment of model performance in the postdevelopment period.

Multimedia Appendix 3 Stratified heterogeneity analysis.

## Abbreviations

AL: active learning
AUC-ROC: area under the receiver operating characteristic curve
CUSUM: cumulative sum
MMD: maximum mean discrepancy
SH: stratified heterogeneity
UDA: unsupervised domain adaptation
