# Supplementary material for: A Comprehensive Drift-Adaptive Framework for Sustaining Model Performance in COVID-19 Detection From Dynamic Cough Audio Data: Model Development and Validation
Source: J Med Internet Res. 2025 Jun 3;27:e66919. doi: 10.2196/66919 (PMC12174887; doi:10.2196/66919)
Supplement: Multimedia Appendix 1 [file jmir_v27i1e66919_app1.docx]

# Multimedia Appendix 1: Hyperparameters’ tuning

Tuning referred to hyperparameters related to data stream scanning and drift detection, which are summarized together with their examined values in Table 1.1. The tuning procedure considered hyperparameters used for data stream scanning (window length, overlap between successive windows, minimum batch size) and drift detection (kernel type and reference distribution for the MMD distance calculation and drift and threshold values of the CUSUM algorithm). The consideration of the minimum amount of data in a batch among the investigated hyperparameters enabled the inclusion of sufficient data during periods with a low data acquisition frequency, thus ensuring the robustness of the framework.

*Table 1.1: Investigated hyperparameters’ values of the proposed framework.*

| Hyperparameters | | | Values | |
| --- | --- | --- | --- | --- |
| Data stream scanning | Time window length | {7, 10, 14} days | |  |
|  | Time windows overlap | {70%, 60%, 50%, 40%, 30%, 20%, 10%, 0%} | |  |
|  | Minimum batch size | {0, 10, 20, 30, …, max batch size} | |  |
| Drift detection mechanism | CUSUM Drift | {0.2, 0.3, 0.4, 0.5} | |  |
|  | CUSUM Threshold | {0.5, 0.6, 0.7, 0.8, 0.9, 1} | |  |
|  | Reference distribution | {Positive data, Negative data, All data} | |  |
|  | MMD kernel | {Linear, Polynomial, Gaussian} | |  |

An offline nested approach, aiming at emulating the real-time operation scenario of the framework, was adopted for fine-tuning the framework’s hyperparameters. In this context, a subset of the development data (70%), namely D-H set, was used for the creation of a baseline-H model. The D-H set was partitioned into training, validation, and test subsets based on a 60:20:20 split ratio. The rest of the development data (30%), named FT-H set, served for fine-tuning the hyperparameters’ values (Figure 1.1).

| 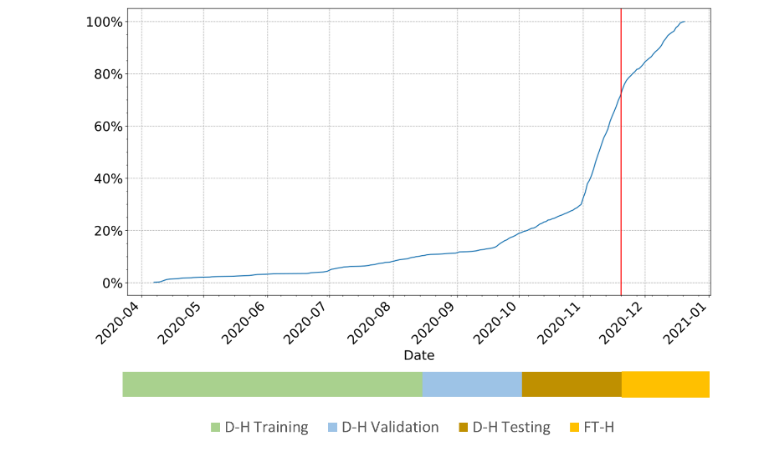 | 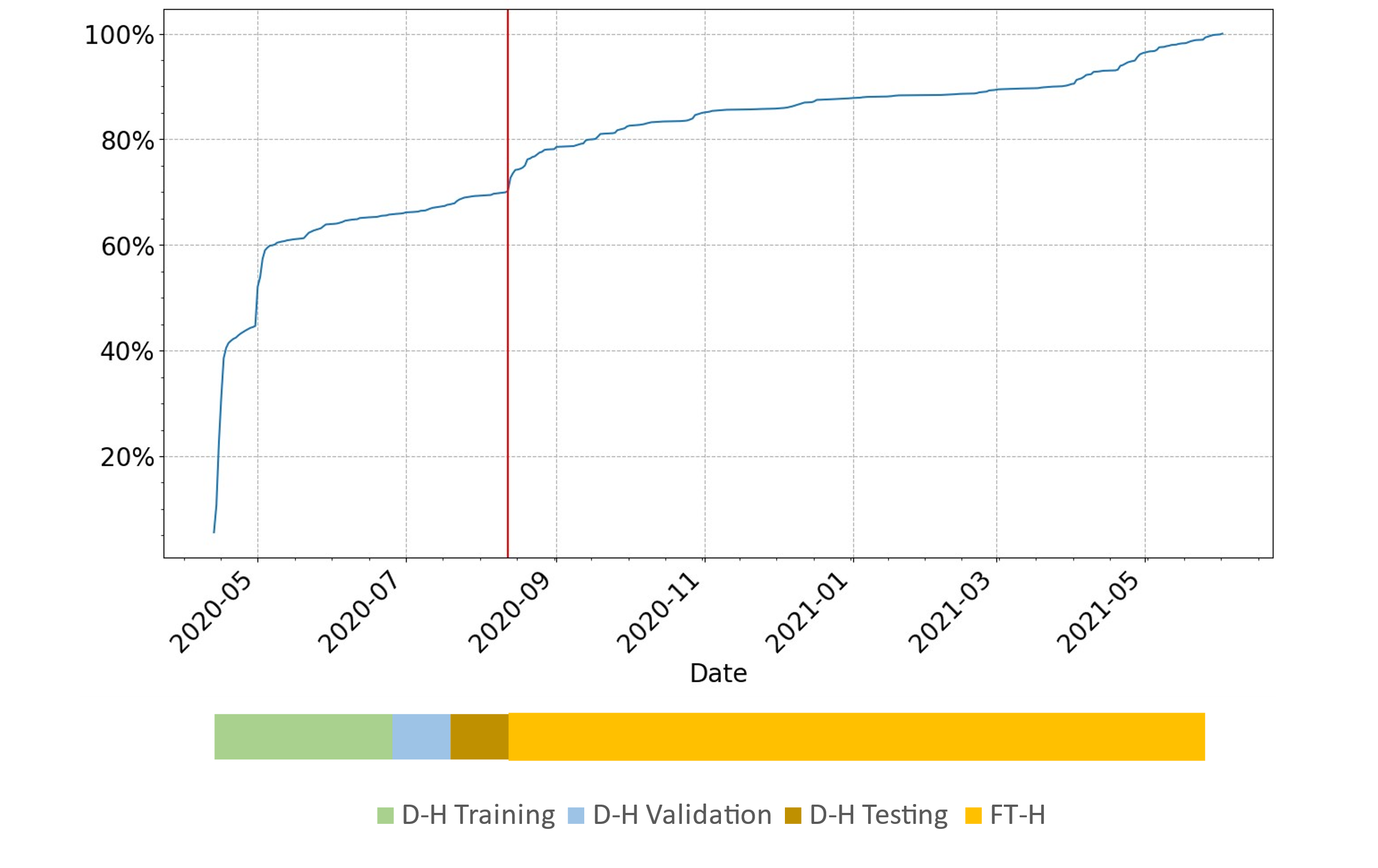 |
| --- | --- |

*Figure 1.1: COVID-19 Sounds (left panel) and COSWARA (right panel) data streams over time for the hyperparameters’ fine-tuning. A 70:30 partition of the development data into D-H and FT-H sets (indicated by a vertical red line) was applied. The D-H set was further partitioned into a training, validation, and test subset using a 60:20:20 ratio.*

The performance of the baseline-H model, measured in terms of the balanced accuracy on the test subset of the D-H set, was used as a benchmark against which the performance of the baseline-H model on the FT-H data batches was assessed. Any batch with performance below the benchmark was considered to be associated with a drift period and was properly labeled. The investigated hyperparameters’ combinations were comparatively assessed in terms of their ability to correctly identify the labeled batches using appropriate metrics (accuracy, sensitivity, and specificity). The adopted offline nested approach is illustrated in Figure 1.2.


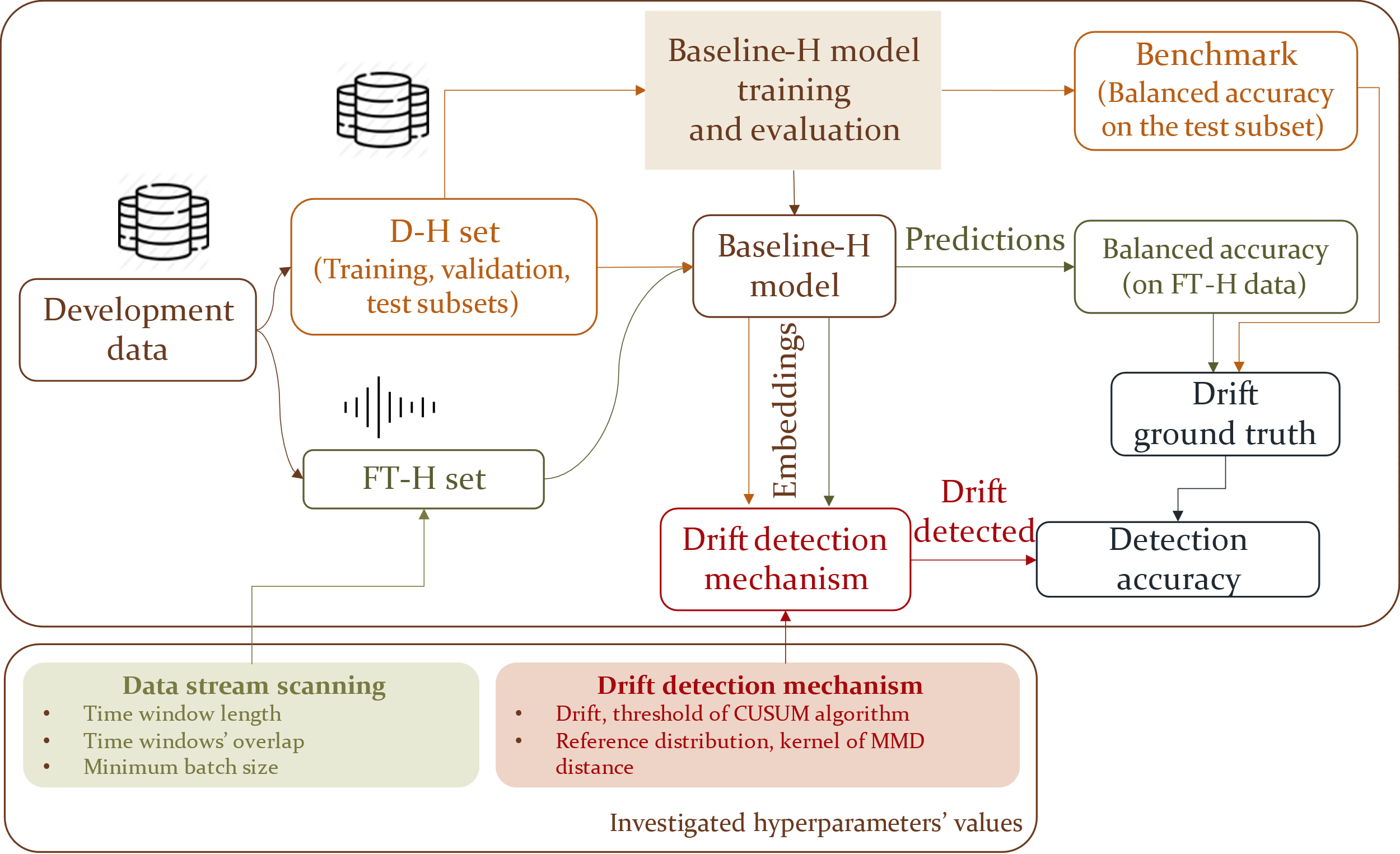


*Figure 1.2: Nested approach for the hyperparameters’ fine-tuning. A 70:30 partition of the development data into D-H and FT-H sets was applied. The D-H set (further partitioned into a training, validation, and test subset using a 60:20:20 ratio) was used for developing a baseline-H model exclusively for hyperparameters’ tuning. The balanced accuracy score on the test subset of the D-H set served as a benchmark. The FT-H set was used for fine-tuning the framework’s hyperparameters to accurately detect batches where the model's performance fell below the benchmark.*

The balanced accuracy was selected for assessing model performance due to its ability to effectively capture the model’s discriminative ability in the presence of class imbalance. Moreover, balanced accuracy was able to provide an appropriate measure even in the case of batches with a limited number of data instances, where the AUC score could not be applied.

Table 1.2 and Table 1.3 depict the top 10 hyperparameters’ combinations based on the obtained accuracy for the COVID-19 Sounds and COSWARA cases, respectively. In the context of COVID-19 Sounds, the application of a 7-day window and a 3-day overlap between successive windows resulted in the highest accuracy score. The use of the total population of the development period as reference distribution and the Polynomial kernel for the MMD distance calculation along with a minimum batch size of 40 data samples led to the best performance. The CUSUM algorithm's drift and threshold values were set at 0.2 and 0.5, respectively, which corresponded to a tolerance range of 20% increase between successive batches and an accumulated increase of 50% beyond the tolerance range as optimal for generating alerts towards accurate detections.

In the case of COSWARA, the configuration including a 10-day window, no overlap between successive batches, and the total population of the development period as the reference distribution yielded the best results. In all top-performing combinations, a minimum of 20 data samples per batch was employed. Different combinations with variations of the CUSUM parameters and of the kernel used for the MMD distance calculation achieved similar optimal performance and triggered the same alerts. Based on this remark and in order to enhance the sensitivity of the CUSUM algorithm, the configuration utilizing the lowest values for the drift and threshold parameters, was selected.

*Table 1.2:* *The top ten hyperparameters’ combinations in the case of the Covid-19 Sounds dataset. Detection accuracy, sensitivity, and specificity were used to evaluate the framework’s ability to correctly identify the batches where the model’s performance fell below the benchmark of the development period. The selected optimal combination is highlighted in bold.*

| Time window length (days) | Time windows overlap | Minimum batch size | CUSUM Drift | CUSUM Threshold | Reference distribution | MMD kernel | Accuracy | Sensitivity | Specificity |
| --- | --- | --- | --- | --- | --- | --- | --- | --- | --- |
| **7** | **60%** | **40** | **0.2** | **0.5** | **All data** | **Polynomial** | **0.80** | **0.66** | **1.0** |
| 7 | 40% | - | 0.4 | 0.8 | Positive | Polynomial | 0.75 | 0.33 | 1.0 |
| 7 | 40% | - | 0.3 | 0.7 | Positive | Gaussian | 0.75 | 0.33 | 1.0 |
| 7 | 40% | - | 0.3 | 0.8 | Positive | Gaussian | 0.75 | 0.33 | 1.0 |
| 7 | 40% | - | 0.4 | 0.5 | Positive | Gaussian | 0.75 | 0.33 | 1.0 |
| 7 | 40% | - | 0.4 | 0.6 | Positive | Gaussian | 0.75 | 0.33 | 1.0 |
| 7 | 40% | - | 0.4 | 0.7 | Positive | Gaussian | 0.75 | 0.33 | 1.0 |
| 7 | 40% | - | 0.5 | 0.5 | Positive | Gaussian | 0.75 | 0.33 | 1.0 |
| 7 | 40% | - | 0.5 | 0.6 | Positive | Gaussian | 0.75 | 0.33 | 1.0 |
| 7 | 40% | - | 0.2 | 0.5 | Positive | Polynomial | 0.75 | 0.33 | 1.0 |

Given that the proposed framework operates in a dynamically evolving manner, the performance evaluation of the drift detection mechanism during the post-development period is discussed in the next sections, along with the results obtained by applying the two different adaptation modules.

*Table 1.3: The top ten hyperparameters’ combinations in the case of the COSWARA dataset. Detection accuracy, sensitivity, and specificity were used to evaluate the framework’s ability to correctly identify the batches where the model’s performance fell below the benchmark of the development period. The selected optimal combination is highlighted in bold.*

| Time window length (days) | Time windows overlap | Minimum batch size | CUSUM Drift | CUSUM Threshold | Reference distribution | MMD kernel | Accuracy | Sensitivity | Specificity |
| --- | --- | --- | --- | --- | --- | --- | --- | --- | --- |
| 10 | 0% | 20 | 0.3 | 0.7 | All data | Polynomial | 0.86 | 0.86 | 0.81 |
| 10 | 0% | 20 | 0.2 | 0.9 | All data | Linear | 0.86 | 0.86 | 0.81 |
| 10 | 0% | 20 | 0.3 | 0.9 | All data | Polynomial | 0.86 | 0.86 | 0.81 |
| **10** | **0%** | **20** | **0.2** | **0.7** | **All data** | **Linear** | **0.86** | **0.86** | **0.81** |
| 10 | 0% | 20 | 0.2 | 0.8 | All data | Linear | 0.86 | 0.86 | 0.81 |
| 10 | 0% | 20 | 0.3 | 0.8 | All data | Polynomial | 0.86 | 0.86 | 0.81 |
| 10 | 0% | 20 | 0.2 | 1.0 | All data | Linear | 0.86 | 0.86 | 0.81 |
| 7 | 0% | 20 | 0.2 | 0.5 | All data | Linear | 0.83 | 0.83 | 0.75 |
| 14 | 90% | 20 | 0.3 | 0.6 | All data | Polynomial | 0.82 | 0.81 | 0.75 |
| 14 | 90% | 20 | 0.2 | 0.5 | All data | Polynomial | 0.82 | 0.81 | 0.62 |
