# Supplementary material for: A Comprehensive Drift-Adaptive Framework for Sustaining Model Performance in COVID-19 Detection From Dynamic Cough Audio Data: Model Development and Validation
Source: J Med Internet Res. 2025 Jun 3;27:e66919. doi: 10.2196/66919 (PMC12174887; doi:10.2196/66919)
Supplement: Multimedia Appendix 2 [file jmir_v27i1e66919_app2.docx]

# Multimedia Appendix 2: Assessment of model performance in the post-development period

**Unsupervised** **domain adaptation**

A comparative assessment of the model’s performance before and after each adaptation phase using the UDA approach was carried out. The performance of the baseline model, measured in terms of the balanced accuracy on the test subset of the development period, served as a benchmark for the evaluation of the model’s performance after each adaptation period. For the COVID-19 Sounds dataset, the obtained balanced accuracy percentage change between the baseline model and the model after each adaptation phase is presented in Figure 2.1. It is evident that immediately after each alert period, the model's performance exhibited a considerable improvement, demonstrating the effectiveness of the proposed approach in mitigating the impact of concept drift. More specifically, following the first adaptation, the model showed an improved performance by up to 6% in terms of the balanced accuracy, which was maintained for four consequent batches (batches 2-5) before a second alert was triggered. Following the second adaptation, the model consistently outperformed the baseline model by up to 8%. After the third adaptation, the model enhanced its performance by more than 4%, remaining superior to the baseline model until the next drift detection, when the fourth adaptation led to a performance improvement of up to 15%, which was maintained across ten batches (batches 19-28). After the fifth adaptation, the model exhibited a maximum performance increase of up to 24% and continued outperforming the baseline model until the end of the data stream. It is noteworthy that by correctly identifying periods of drift, the drift detection mechanism efficiently prevented the degradation of the model’s performance in a timely manner while also contributing to sustaining the model’s performance closer to the benchmark established during its development period.


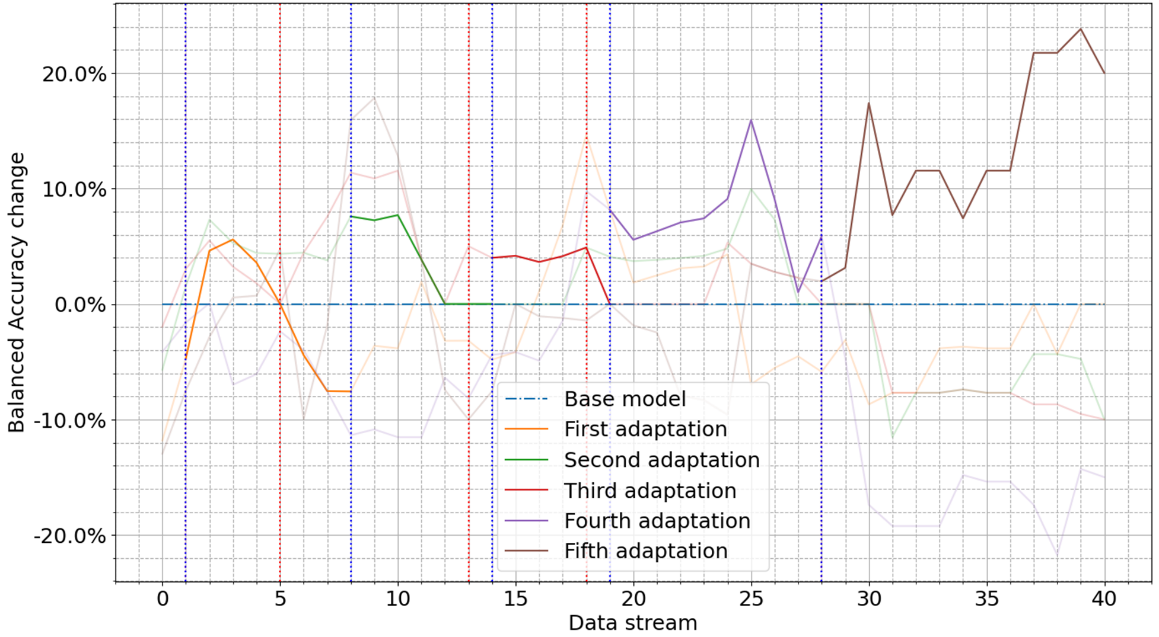


*Figure 2.1: Percentage change of the obtained balanced accuracy score across the data batches of the entire post-development period of the COVID-19 Sounds dataset using UDA. Vertical red and blue dotted lines indicate the start and end of each alert period.*

In Figure 2.2, the discrimination performance of the adapted models is compared with that of the baseline model over their respective operational periods in terms of various performance metrics. It can be seen that after the first and fourth adaptation, each adapted model exhibited decreased performance in terms of the AUC, accuracy, sensitivity, and F1 score, but performed equally well or better than the baseline model in terms of the balanced accuracy, precision, and specificity. The other adaptations (second, third, fifth) resulted in a consistent and gradually increasing performance improvement according to all used evaluation metrics.


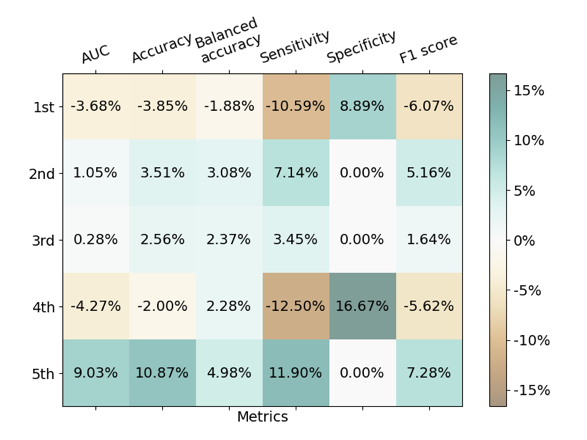


*Figure 2.2: UDA approach on COVID-19 Sounds dataset. Percentage change of model performance after each adaptation (1st – 5th) with respect to the baseline model.*

Regarding the COSWARA dataset, it is observed that the first adaptation led to a significant yet short-lasting decline in the model's performance, which persisted for two batches following the alert (until batch 4), as depicted in Figure 2.3. Subsequently, the model exhibited a consistent and steady performance improvement, reaching up to a 20% increase compared to the baseline model's performance. Following the second adaptation, the model's performance presented a decline lasting for six batches after the alert, eventually returning to the baseline model's performance level. After the third adaptation, the model displayed a performance drop for one batch, followed by an increase of up to 10% that lasted for two batches. The fourth adaptation resulted in enhanced performance, demonstrating an improvement of up to 15% compared to the baseline model.


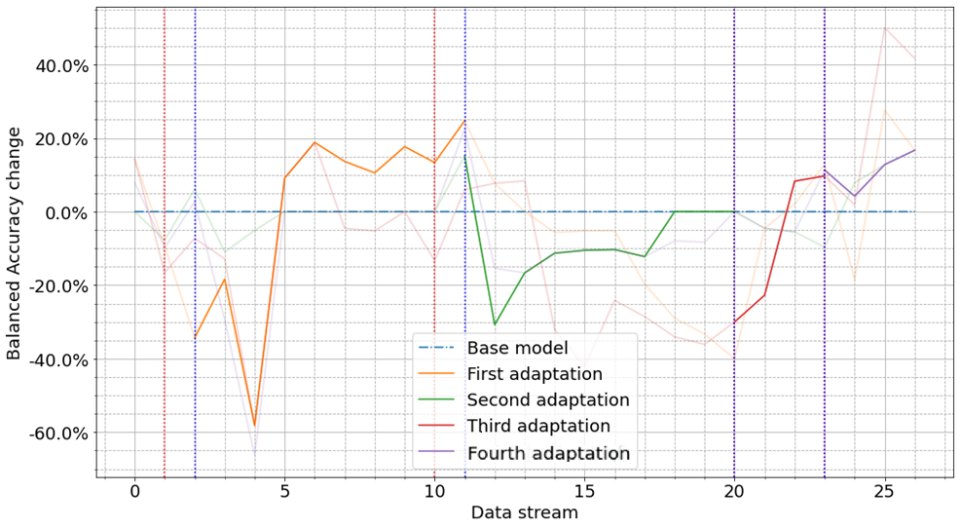


*Figure 2.3: Percentage change of the obtained balanced accuracy score across the data batches of the entire post-development period of the COSWARA dataset using UDA. Vertical red and blue dotted lines indicate the start and end of each alert period.*

Although in the case of the COSWARA dataset, the UDA method was particularly challenged in maintaining the model's performance, the drift detection mechanism consistently demonstrated effectiveness by generating timely alerts related to anticipated declines in the model's performance. Notably, the first alert was triggered during a phase where the model's performance was gradually decreasing, although it still remained above the benchmark. The second alert was triggered immediately after a performance drop below the benchmark level, preventing an even steeper decline. Similarly, the third and fourth alerts were produced amid significant performance deterioration, aligning precisely with instances where the model fell below the benchmark level, thus indicating the robustness of the drift detection mechanism. The comparative assessment of the performance of the baseline model and the model after each adaptation (Figure 2.4) demonstrated that the implemented adaptations resulted in varying performance in terms of the different evaluation metrics.


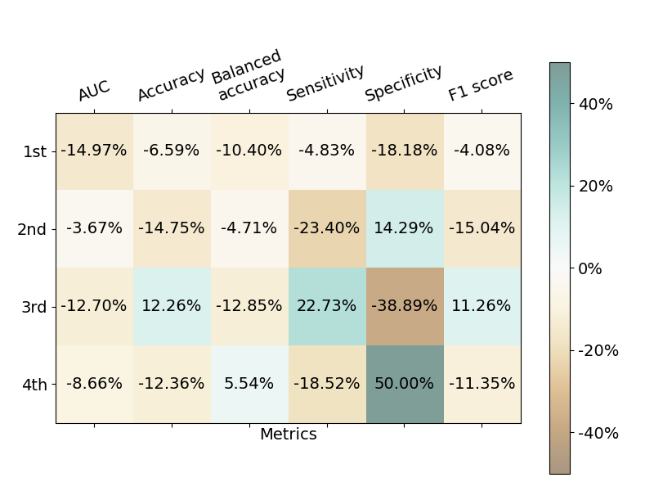


*Figure 2.4: UDA approach on COSWARA dataset. Percentage change of model performance after each adaptation (1st – 4th) with respect to the baseline model.*

**Active learning**

The proposed AL approach was evaluated by comparing the performance of the model after each AL-based retraining phase with that of the baseline model as well as the model following retraining using random sampling. Looking at the COVID-19 Sounds dataset, Figure 2.5 demonstrates the observed balanced accuracy score across the entire data stream, indicating a substantial and lasting improvement with respect to the baseline model following each adaptation. After the first adaptation, the model exhibited improved performance by up to 20% until the second alert, while a similar improvement was achieved following the second adaptation. After the third adaptation, the model’s performance demonstrated a significant increase of up to 30%, sustained over a broad period of 15 batches. After the fourth adaptation, the model showcased outstanding performance, surpassing a 95% balanced accuracy score while achieving an improvement of up to 25% compared to the baseline model.


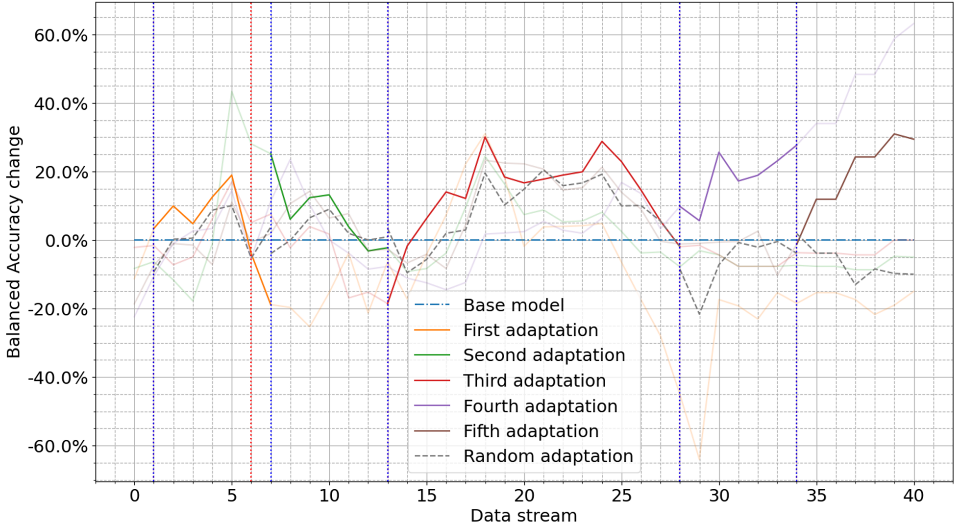


*Figure 2.5: Percentage change of the obtained balanced accuracy score across the data batches of the entire post-development period of the COVID-19 Sounds dataset using AL. Vertical red and blue dotted lines indicate the start and end of each alert period.*

| 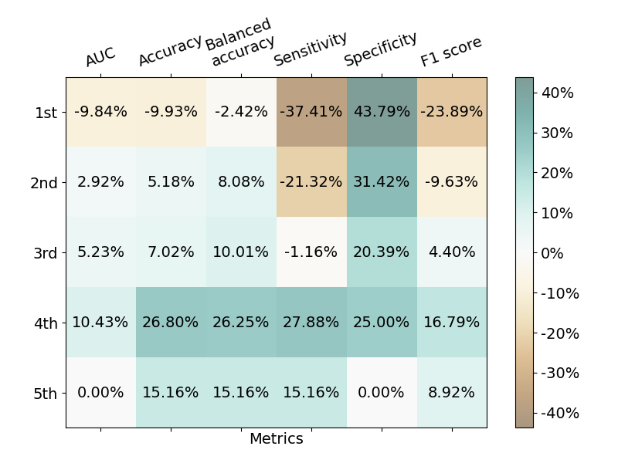  (a) | 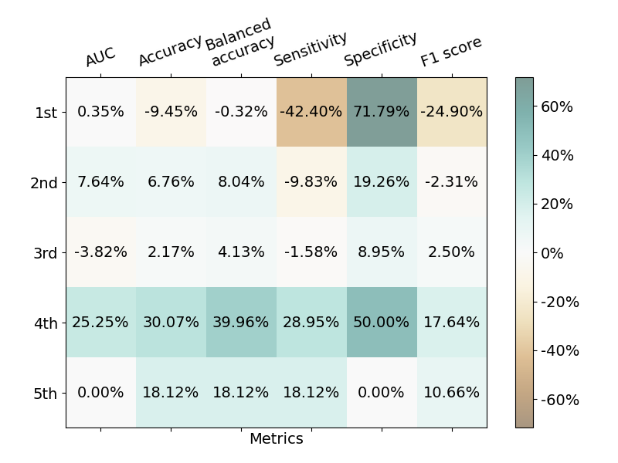  (b) |
| --- | --- |

*Figure 2.6: AL approach on the COVID-19 Sounds dataset. Percentage change of model performance after each adaptation (1st – 5th) with respect to the baseline model (left panel) and the model after retraining through random sampling (right panel).*

The performance comparison with the random sampling approach highlighted the superiority of the proposed AL approach, indicating its ability to identify informative data. Specifically, the random sampling approach outperformed AL only in four batches (i.e., batches 7, 12, 13, and 21) out of the 41 batches of the data stream. AL significantly outperformed the random sampling approach in all other cases. Similar conclusions can be drawn from Figure 2.6, where it can be seen that the majority of adaptations based on AL yielded increased performance in terms of all the considered evaluation metrics with respect to the baseline model and the random sampling approach.

In the case of the COSWARA dataset, Figure 2.7 shows that model adaptations led to improved performance during the post-alert periods. Following the first adaptation, there was a marginal increase in the balanced accuracy score, with the model slightly outperforming the baseline model until the subsequent alert. After the second adaptation, a notable enhancement of up to 20% in the balanced accuracy score was observed in the subsequent batches, while the fourth adaptation resulted in performance improvement of up to 25%. In terms of the third and fifth adaptations, although the model's performance exhibited significant enhancement, reaching up to 40% and 60%, respectively, performance fluctuations were also observed, with the model being outperformed by the baseline model in some batches.


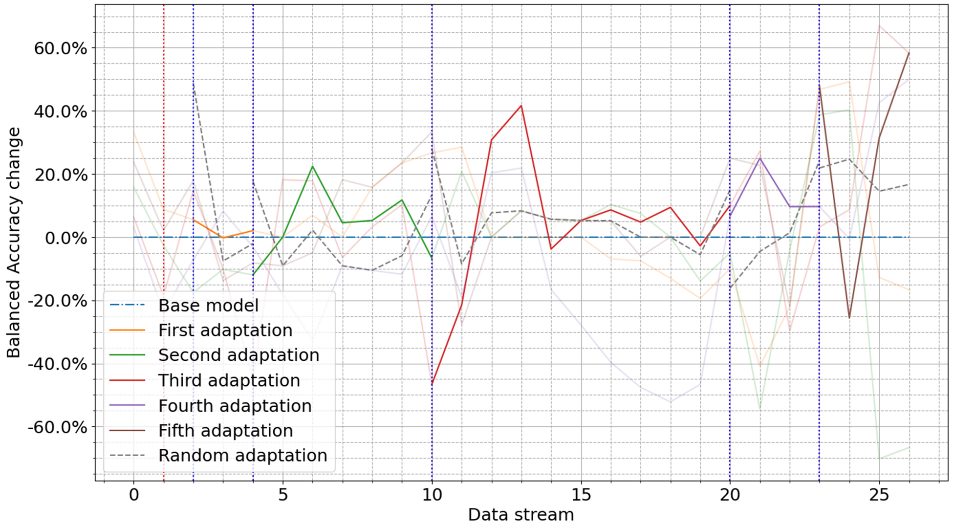


*Figure 2.7: Percentage change of the obtained balanced accuracy score across the data batches of the entire post-development period of the COSWARA dataset using AL. Vertical red and blue dotted lines indicate the start and end of each alert period.*

When comparing the performance of the AL method with the results obtained through random sampling, it becomes evident that the former method demonstrated superior performance throughout the majority of the data batches except for specific instances (within batches 2, 4, 10, 11, 14, 24). This highlighted AL’s efficiency in identifying informative data.

Further insights on the model’s performance after each adaptation, with respect to the baseline model and the model after retraining based on random sampling, can be drawn from Figure 2.8. It can be seen that the AL approach led to an overall improvement in the model's performance over the baseline model, except for certain metrics/adaptations (Figure 2.8(a)). This is evident in the second adaptation, where the model exhibited an improved AUC score and specificity, but inferior performance in terms of the remaining evaluation metrics. The comparison with the random sampling approach (Figure 2.8(b)) revealed a more intricate pattern. The third and fourth adaptations, spanning for more than half of the entire data stream, clearly had a positive impact on the model’s performance. However, the first adaptation resulted in higher sensitivity levels at the cost of lower specificity, while the second and fifth adaptations yielded lower performance.

| 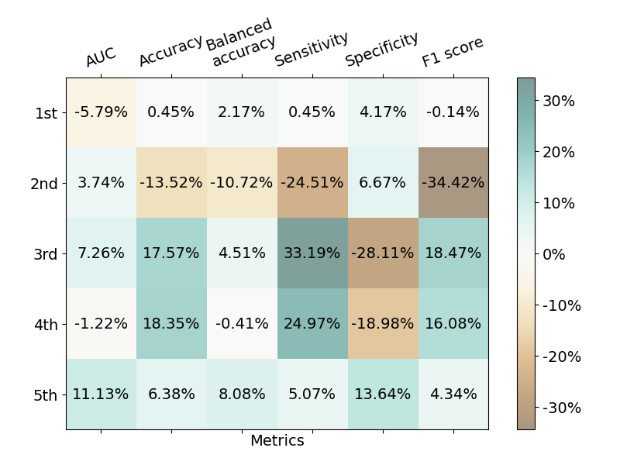  (a) | 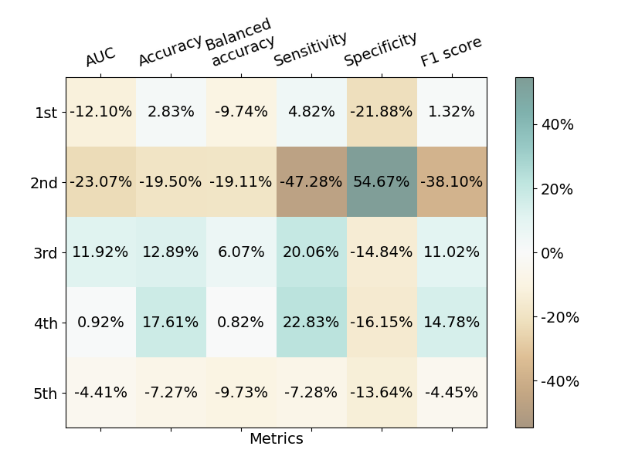  (b) |
| --- | --- |

*Figure 2.8: AL approach on the COSWARA dataset. Percentage change of model performance after each adaptation (1st – 5th) with respect to the baseline model (left panel) and the model after retraining through random sampling (right panel).*
