# Supplementary material for: A Comprehensive Drift-Adaptive Framework for Sustaining Model Performance in COVID-19 Detection From Dynamic Cough Audio Data: Model Development and Validation
Source: J Med Internet Res. 2025 Jun 3;27:e66919. doi: 10.2196/66919 (PMC12174887; doi:10.2196/66919)
Supplement: Multimedia Appendix 3 [file jmir_v27i1e66919_app3.docx]

# Multimedia Appendix 3: Stratified heterogeneity analysis

To further assess the impact of data drift on model performance, a detailed analysis using the q-statistic as a measure of stratified heterogeneity (SH) was conducted. The q-statistic was calculated for the ground truth label (covid-19 presence) determined by different stratification factors: time (batch), age, gender, and their interactions (pairwise and three-way). This enabled measuring the degree of heterogeneity across the dimensions of time, age, and gender, and understanding its variation over time.

The relationship between the degree of SH and model performance was examined by correlating performance variations across the defined strata, based on the considered stratification factors, with the corresponding q-statistic values. A higher q-statistic indicated greater heterogeneity, which could contribute to fluctuations in model performance across different subpopulations.

Moreover, an investigation of the potential correlations between the generated drift alerts by the proposed drift detection mechanism and the degree of SH in the ground truth label was carried out. To this end, a supervised approach (SH-based approach) was employed retrospectively for identifying temporal drifts in the ground truth label distribution based on the statistically significant differences in the q-statistic values between successive batches. In particular, a temporal drift was considered present in batches exhibiting statistically significant differences (P-value<=0.05) in their q-statistic compared to their 3 preceding batches. The identified batches with temporal drifts in the ground truth label distribution were then compared with the drift alerts produced by the proposed unsupervised and real-time drift detection mechanism. This comparison enabled the assessment of possible correlations between shifts in input data distribution and variations in output label heterogeneity. While the proposed drift detection mechanism primarily captures shifts in the data embeddings distribution, the SH-based approach quantifies the heterogeneity of the ground truth labels. By integrating these two perspectives, the analysis provided deeper insights into the interplay between input data distribution shifts and output label heterogeneity.

## COVID-19 Sounds Dataset results

The analysis of the COVID-19 Sounds Dataset revealed that the ground truth label exhibited minimal SH with respect to gender (q = 0.0009) and age (q = 0.011), while SH was moderate in terms of time (q = 0.07), as shown in Table 3.1. However, interactions involving time (batch) increased the degree of SH, with gender-batch yielding a q-statistic of 0.1, age-batch reaching 0.15, and the combination of all three factors increasing the q-statistic to 0.2.

A strong correlation was observed between the obtained q-statistic values and the model performance variability for the defined strata across both baseline and adapted models, with a Pearson Correlation Coefficient (PCC) of 0.96 for the baseline model, 0.95 for the UDA approach, and 0.96 for the AL approach (Table 3.1).

When comparing the drift alerts generated by the proposed drift detection mechanism with the temporal drifts in the ground truth label identified by the SH-based approach, the latter highlighted batches that were close (within two batches or fewer) to those associated with the detected drift alerts, regardless of the adaptation method applied. Specifically, the SH-based approach identified batches corresponding to the second, third, and fifth drift alerts yielded in the case of the UDA-based adapted model (Figure 3.1) and the second, third, fourth, and fifth drift alerts in the case of the AL-based adapted model (Figure 3.2). Notably, in five out of the six batches identified by the SH-based approach, both the UDA- and AL-based adapted models outperformed the baseline model as well as the model adapted using random sampling, with the exception of batches 7 and 13 for UDA and AL, respectively.

Table 3.1: Q-statistic values for the ground truth label of COVID-19 sounds dataset determined by each stratification factor and their interactions. The performance disparity is calculated as the performance (balanced accuracy) variance accross strata. The correlation was calculated between pairs of q-statistic and performance disparity.

| **Stratification factor (Determinant)** | **q-statistic** |  | **Performance variance (Baseline model)** | **Performance variance (UDA)** | **Performance variance (AL)** |
| --- | --- | --- | --- | --- | --- |
| **Time** | 0.076 |  | 0.047 | 0.055 | 0.044 |
| **Age group** | 0.011 |  | 0.031 | 0.026 | 0.026 |
| **Gender** | 0.0009 |  | 0.002 | 0.002 | 0.001 |
| **Time – Age group** | 0.158 |  | 0.147 | 0.155 | 0.156 |
| **Time – Gender** | 0.1 |  | 0.086 | 0.095 | 0.092 |
| **Age group – Gender** | 0.022 |  | 0.058 | 0.075 | 0.054 |
| **Time - Age group - Gender** | 0.202 |  | 0.177 | 0.182 | 0.175 |
|  |  |  |  |  |  |
| **Pearson Correlation Coefficient** |  |  |  |  |  |
| **Baseline model** | 0.96 |  |  |  |  |
| **UDA** | 0.95 |  |  |  |  |
| **AL** | 0.96 |  |  |  |  |


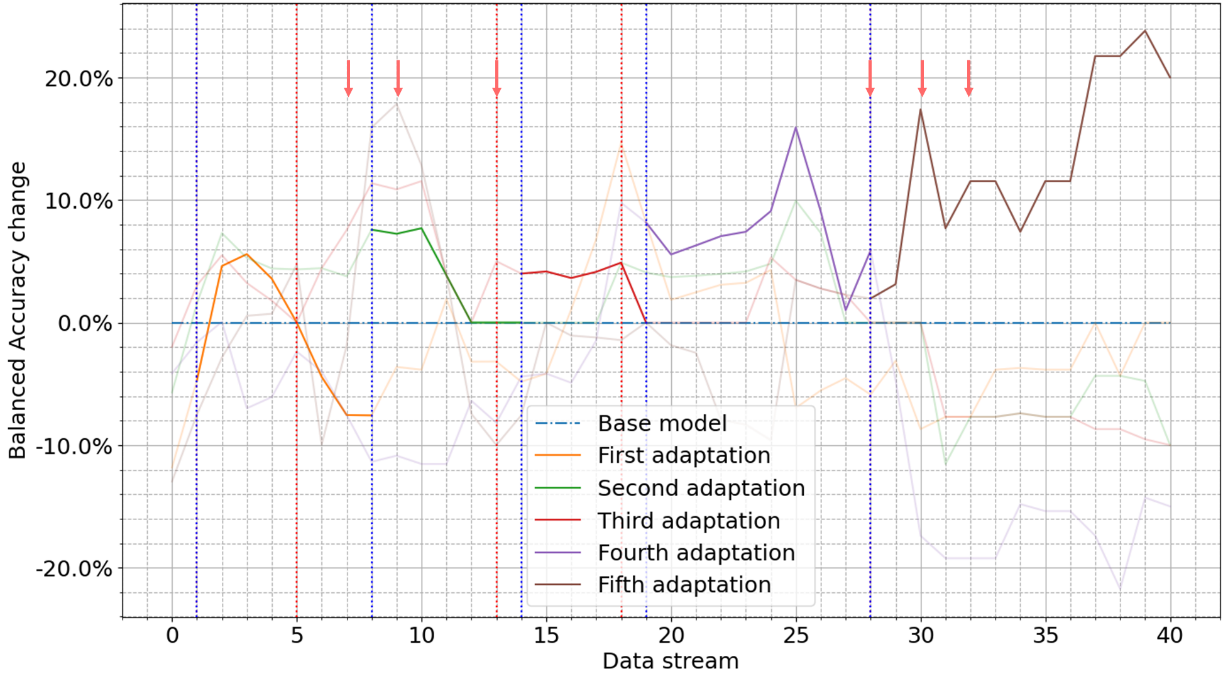


Figure 3.1: Percentage change of the obtained balanced accuracy score across the entire post-development period of the COVID-19 Sounds dataset using UDA. Vertical red and blue dotted lines indicate the start and end of each alert period detected by the proposed drift detector. Red arrows indicate batches with drifts in the ground truth label, detected based on the SH-based approach.


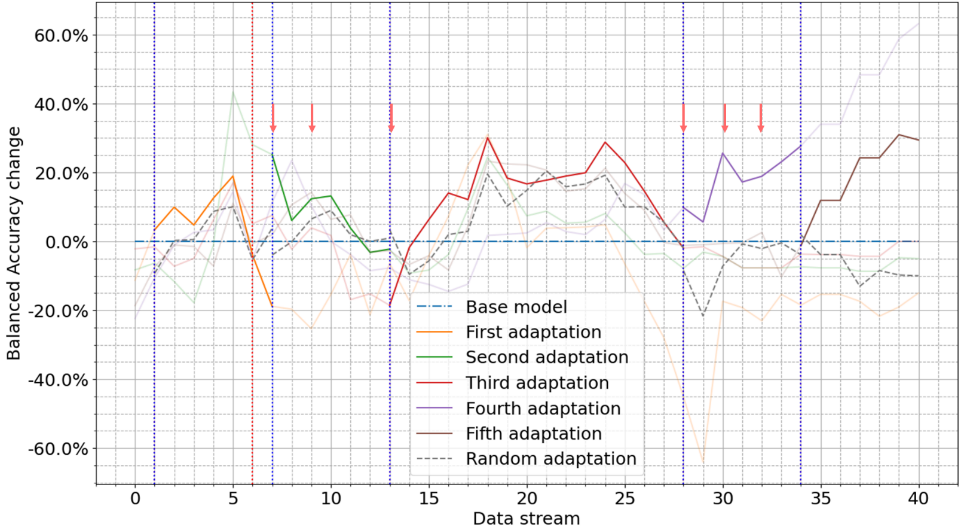


Figure 3.2: Percentage change of the obtained balanced accuracy score across the entire post-development period of the COVID-19 Sounds dataset using AL. Vertical red and blue dotted lines indicate the start and end of each alert period detected by the proposed drift detector. Red arrows indicate batches with drifts in the ground truth label, detected based on the SH-based approach.

## COSWARA Dataset Results

The analysis of the COSWARA Dataset revealed that the ground truth label exhibited low SH in terms of gender (q = 0.019) and age (q = 0.061), while SH was strong with respect to time (q = 0.45), as depicted in Table 3.2. Moreover, the interactions of stratification factors increased the degree of SH, with the combination of all three factors yielding a q-statistic equal to 0.53.

The correlation between the obtained q-statistic values and the model performance variability for the defined strata across both baseline and adapted models, while weaker than that observed in the COVID-19 sounds case, was high, with a PCC value of 0.76 for the baseline model, 0.75 for the UDA approach, and 0.84 for the AL approach (Table 3.2).

In terms of the comparison between the drift alerts by drift detection mechanism and the temporal drifts in the ground truth label identified by the SH-based approach, the SH-based approach highlighted batches that were close (within three batches or fewer) to those associated with the detected drift alerts for both adaptation methods. Specifically, the batches identified by the SH-based approach corresponded to the first, second, and fourth drift alerts yielded by the UDA-based adapted model (Figure 3.3) and the first, third, and fifth alerts in the case of the AL-based model (Figure 3.4). Moreover, while the first two batches identified by the SH-based approach preceded the drift alerts generated by the drift detection mechanism, in five of the remaining seven batches identified by the SH-based approach, the UDA-based adapted model outperformed the baseline model, while the AL-based model’s performance was superior to that of the adapted model using random sampling in four out of the seven identified batches.

Table 3.2: Q-statistic values for the ground truth label of COSWARA dataset determined by each stratification factor and their interactions. The performance disparity is calculated as the performance (balanced accuracy) variance accross strata. The correlation was calculated between pairs of q-statistic and performance disparity.

| **Stratification Factor (Determinant)** | **q-statistic** |  | **Performance variance (Baseline model)** | **Performance variance (UDA)** | **Performance variance (AL)** |
| --- | --- | --- | --- | --- | --- |
| **Time** | 0.458 |  | 0.042 | 0.043 | 0.055 |
| **Age group** | 0.061 |  | 0.016 | 0.017 | 0.012 |
| **Gender** | 0.019 |  | 0.05 | 0.051 | 0.042 |
| **Time – Age group** | 0.504 |  | 0.126 | 0.13 | 0.117 |
| **Time – Gender** | 0.472 |  | 0.078 | 0.079 | 0.074 |
| **Age group – Gender** | 0.082 |  | 0.031 | 0.032 | 0.028 |
| **Time - Age group - Gender** | 0.531 |  | 0.145 | 0.148 | 0.133 |
|  |  |  |  |  |  |
| **Pearson Correlation Coefficient** |  |  |  |  |  |
| **Baseline model** | 0.76 |  |  |  |  |
| **UDA** | 0.75 |  |  |  |  |
| **AL** | 0.84 |  |  |  |  |


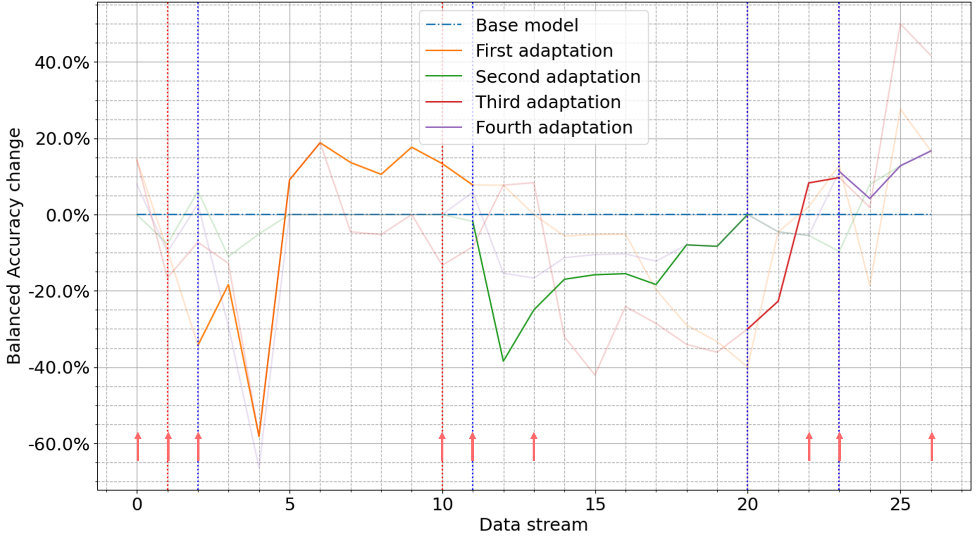


Figure 3.3: Percentage change of the obtained balanced accuracy score across the entire post-development period of the COSWARA dataset using UDA. Vertical red and blue dotted lines indicate the start and end of each alert period detected by the proposed drift detector. Red arrows indicate batches with drifts in the ground truth label, detected based on the SH-based approach.


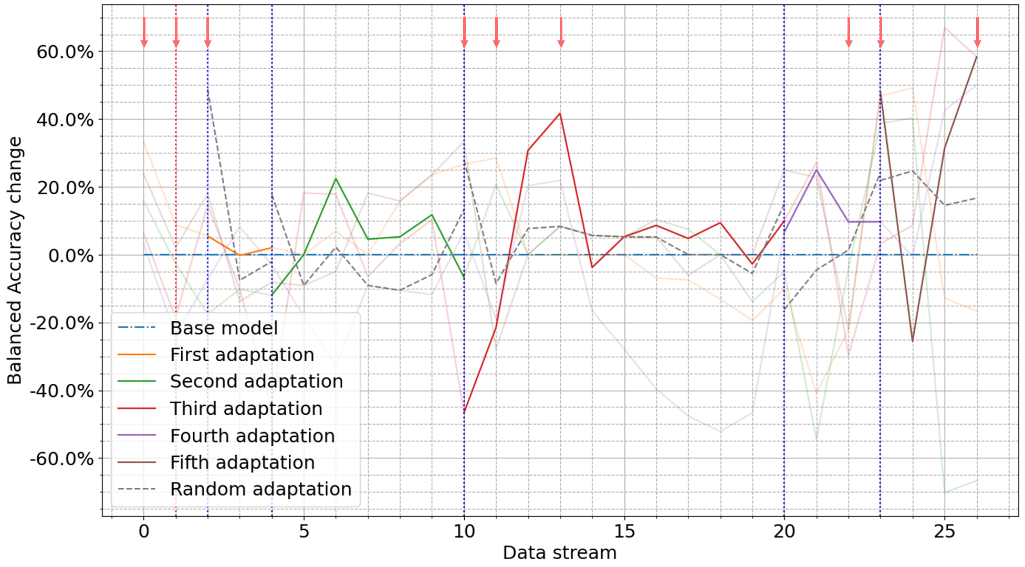


Figure 3.4: Percentage change of the obtained balanced accuracy score across the entire post-development period of the COSWARA dataset using AL. Vertical red and blue dotted lines indicate the start and end of each alert period detected by the proposed drift detector. Red arrows indicate batches with drifts in the ground truth label, detected based on the SH-based approach.
